# Piezoelectric characteristics of PVA/DL-alanine polycrystals in d_33_ mode

**DOI:** 10.1016/j.isci.2022.105768

**Published:** 2022-12-08

**Authors:** Buil Jeon, Dongsoo Han, Giwan Yoon

**Affiliations:** 1 School of Electrical Engineering, Korea Advanced Institute of Science and Technology, 291 Daehak-ro, Yuseong-gu, Daejeon 34141, South Korea; 2 School of Computing, Korea Advanced Institute of Science and Technology, 291 Daehak-ro, Yuseong-gu, Daejeon 34141, South Korea

**Keywords:** Mechanical property, Electrical property, Materials characterization techniques

## Abstract

In this study, polyvinyl alcohol (PVA)-mixed DL-alanine (PVA/DL-alanine) polycrystals are fabricated, and their piezoelectric characteristics in the d_33_ mode are investigated. The d_33_ piezoelectric coefficients of the PVA/DL-alanine polycrystals are found to increase with an increase in the weight ratio of DL-alanine, and the PVA/DL-alanine polycrystal composed of PVA and DL-alanine in a weight ratio of 1:3 exhibits a d_33_ of ∼5 pC/N. The piezoelectric characteristics of the PVA/DL-alanine polycrystals are discussed in terms of the crystal structure by employing scanning electron microscopy and X-ray diffraction analyses. To confirm the piezoelectric performance of the polycrystals, the piezoelectric voltages of a piezoelectric device composed of a single layer of ZnO thin film and heterostructured devices consisting of a layer of PVA/DL-alanine polycrystal and a ZnO thin film layer are measured and compared. This study presents PVA/DL-alanine polycrystals as a potential piezoelectric material for bio-friendly piezoelectric-device applications.

## Highlights

• PVA/DL-alanine polycrystals show piezoelectric characteristics in the d_33_ mode
• PVA/DL-alanine polycrystals consist of DL-alanine with c-axis aligned to the 3-axis
• Piezoelectricity of PVA/DL-alanine polycrystals depends on the alignment of DL-alanine

## Graphical abstract

**Figure:**
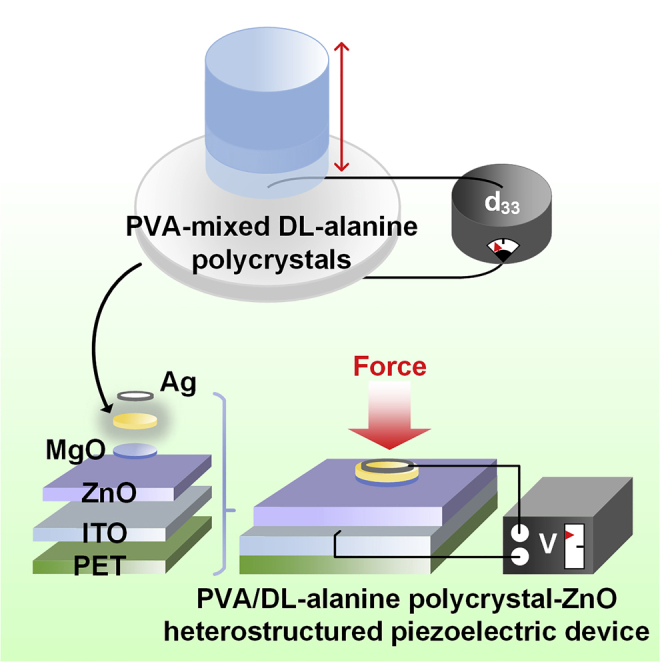

## Introduction

In the field of piezoelectric effect, piezoelectric materials are mainly derived from inorganic materials such as zinc oxide (ZnO),^1^^,^^2^ aluminum nitride (AlN),^2^^,^^3^ gallium nitride (GaN),^4^^,^^5^ barium titanate (BaTiO_3_),^6^ and Pb(Mg_1/3_Nb_2/3_)O_3_-PbTiO_3_ (PMN-PT).^7^^,^^8^ However, recently, in addition to conventional piezoelectric materials, amino acids, which could be crystallized in non-centrosymmetric crystal structures, have attracted increasing attention because piezoelectric amino acids are bio-friendly and biodegradable with advantages such as a low fabrication temperature and low cost.^9^^,^^10^^,^^11^^,^^12^^,^^13^^,^^14^^,^^15^^,^^16^^,^^17^

Among various piezoelectric amino acids, glycine, a polymorphic amino acid having three different crystal structures, α, β, and γ, has been extensively studied as a piezoelectric material, because of the piezoelectric property of β- and γ-glycine being comparable to that of inorganic piezoelectric materials.^11^^,^^12^^,^^13^^,^^14^ However, despite the promising piezoelectric characteristics of β- and γ-glycine, using glycine as a practical piezoelectric material seems not easy, because β-glycine is not only unstable in air but also rarely obtained under general conditions, and γ-glycine could be obtained from metal salt-dissolved solutions with meticulously controlled pH conditions.^18^^,^^19^^,^^20^^,^^21^^,^^22^^,^^23^

Consequently, the polymorphism of piezoelectric amino acids, as in the case of glycine, indicates that polymorphic amino acids could be unsuitable for use as piezoelectric materials because of the uncertainty of piezoelectricity depending on their crystal structures. Therefore, it is suggested that non-polymorphic piezoelectric amino acids could be more advantageous as piezoelectric materials, and in this study, DL-alanine, a non-polymorphic piezoelectric amino acid, was investigated as a piezoelectric material.^23^ DL-alanine, covered in this study, was crystallized from a solution mixed with polyvinyl alcohol (PVA). This is because pure DL-alanine crystals were brittle even by small impacts or forces, whereas the PVA-mixed DL-alanine (PVA/DL-alanine) polycrystals exhibited better durability with decent moldability, compared to those crystallized from the solution without PVA. The piezoelectric energy-harvesting performance of the PVA/DL-alanine polycrystals was evaluated by measuring the d_33_ piezoelectric coefficient. The PVA/DL-alanine polycrystal with a low DL-alanine concentration, in which the weight ratio of PVA to DL-alanine is 1:0.2, showed an insignificant d_33_ coefficient. However, the d_33_ coefficient of the PVA/DL-alanine polycrystals tended to increase as the weight ratio of DL-alanine, constituting the polycrystals, increased, indicating that the piezoelectric characteristics of the PVA/DL-alanine polycrystals originate from DL-alanine. In particular, the PVA/DL-alanine polycrystal containing PVA and DL-alanine in a weight ratio of 1:3 exhibited a d_33_ of ∼5 pC/N on average, validating that the PVA/DL-alanine polycrystals exhibit a piezoelectric performance comparable to that of γ-glycine.^12^^,^^13^ The crystal structures of the PVA/DL-alanine polycrystals were analyzed mainly by employing scanning electron microscopy (SEM), X-ray diffraction (XRD), and Fourier transform infrared (FT-IR) spectroscopy. Based on these analyses, the piezoelectric characteristics of the polycrystals in the d_33_ mode are discussed, and the piezoelectricity development mechanism of the PVA/DL-alanine polycrystals in the d_33_ mode is proposed. In addition, heterostructured piezoelectric energy harvesters composed of a PVA/DL-alanine polycrystal layer and a ZnO thin film were fabricated, and their piezoelectric open-circuit voltages were measured in the d_33_ mode to substantiate the PVA/DL-alanine polycrystals as potential amino-acid-based piezoelectric materials. This study presents the feasibility of using PVA/DL-alanine polycrystals as potential piezoelectric materials.

## Results

### d_33_ piezoelectric coefficient of the PVA/DL-alanine polycrystals

The piezoelectric characteristics of materials are determined by how many piezoelectric charges are developed on their surfaces by mechanical deformation, such as stress and strain. In other words, it could be considered that the more piezoelectric charges are developed, the better the piezoelectric characteristics are. Accordingly, in this study, to evaluate the piezoelectric characteristics of PVA/DL-alanine polycrystals and investigate their correlation with the weight ratio of PVA and DL-alanine, the d_33_ piezoelectric coefficients of PVA/DL-alanine polycrystals were measured.

The piezoelectric coefficient, d_ij_, indicates the charges per Newton developed at the surface of piezeoelctric materials, when a force is applied to them, as can be determined from its unit, C/N (generally, pC/N is used). The direction of the force applied to piezoelectric materials and the direction of the polarization caused by the piezoelectric charge resulting from the force are indicated by subscripts j and i of the coefficient, respectively; for a normal force, subscript j is represented by the axis of the Cartesian coordinate.^10^^,^^15^^,^^24^

Therefore, the d_33_ piezoelectric coefficient can be considered to represent the induced charges per Newton causing the output (i.e., piezoelectric voltage and current) in a direction parallel to the 3-axis when a force is applied to the piezoelectric material in the 3-axis direction, and the 3-axis is assumed to be the axis parallel to the direction perpendicular to the bottom surface of piezoelectric materials in general. Consequently, d_33_ indicates the piezoelectric charges induced at the top and bottom surfaces of the piezoelectric materials pressed vertically in the 3-axis direction, and thus, the d_33_ piezoelectric coefficient is used to represent the piezoelectric performance of materials. For this reason, therefore, d_33_ piezoelectric coefficient has been used as an index of piezoelectric performance in many studies, and also in this study, the piezoelectric performance of PVA/DL-alanine polycrystals was evaluated and compared in terms of their d_33_ piezoelectric coefficients.

**Figure 1:**
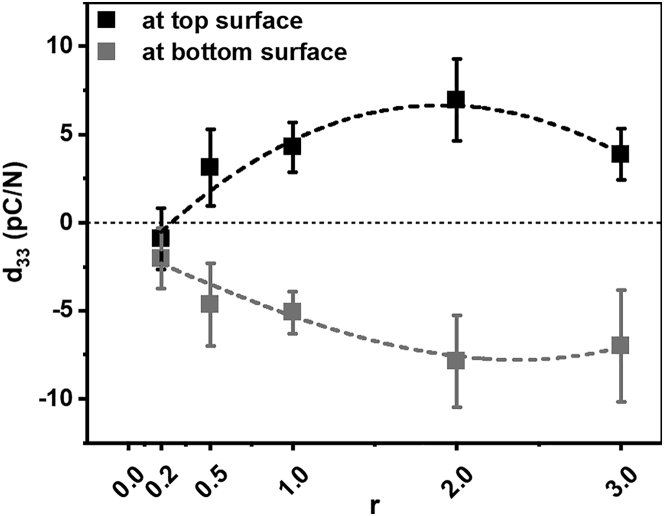
d_33_ piezoelectric coefficients of the PVA/DL-alanine polycrystals d_33_ piezoelectric coefficients at the top (▪) and bottom () surfaces of PVA/DL-alanine polycrystals with r values of 0.2, 0.5, 1, 2, and 3.

Figure 1 shows the d_33_ piezoelectric coefficient of PVA/DL-alanine polycrystals composed of five different weight ratios between PVA and DL-alanine notated as r (i.e., relative weight ratio of DL-alanine to PVA). The details of the measurement of the d_33_ piezoelectric coefficient of the PVA/DL-alanine polycrystals are provided in the STAR Methods section (see method details). The d_33_ piezoelectric coefficient of each PVA/DL-alanine polycrystal was measured ten times on its top and bottom surfaces, respectively, by using a commercial d_33_ meter employing the Berlincourt method. Then, the average value of the d_33_ measured at each surface of the polycrystals was calculated together with the standard deviation.^12^^,^^13^ In Figure 1, it can be confirmed that the d_33_ piezoelectric coefficients measured at the top surfaces of the PVA/DL-alanine polycrystals are positive, except for the polycrystal with an r of 0.2. On the other hand, the d_33_ piezoelectric coefficients measured at the bottom surface are negative for all polycrystals. In other words, except for the polycrystal with an r of 0.2, the polarity of the d_33_ coefficients measured at the top and the bottom surfaces of the PVA/DL-alanine polycrystals is opposite, which indicates that the top and the bottom surfaces of the polycrystals by the applied force are oppositely charged due to the piezoelectric characteristics of the polycrystals.

Accordingly, it can be seen that the PVA/DL-alanine polycrystals whose polarity of the d_33_ is opposite at the top and the bottom surfaces have an effective d_33_ piezoelectric response according to a force whose direction is parallel to the 3-axis, i.e., thickness direction, whereas the PVA/DL-alanine polycrystal with an r of 0.2, exhibiting a negative d_33_ of approximately −2 pC/N at both the top and bottom surfaces, has an ineffective piezoelectric energy-harvesting property in the d_33_ mode,^13^^,^^15^ which seems to be due to the low DL-alanine concentration of the polycrystal with an r of 0.2.

The d_33_ of the PVA/DL-alanine polycrystal with an r of 0.5 is about 2.5 pC/N at the top surface and −5 pC/N at the bottom surface. The d_33_ of the PVA/DL-alanine polycrystal with an r of 1 is 4 and ‒5 pC/N at its top and bottom surfaces, respectively, and it can be confirmed that the d_33_ becomes approximately 7 and ‒7.5 pC/N at the top and bottom surfaces of the PVA/DL-alanine polycrystal with an r of 2. In other words, it was observed that the d_33_ coefficient at the top and bottom surfaces of the PVA/DL-alanine polycrystals is proportional to the relative weight ratio of DL-alanine to PVA (i.e., r) constituting the polycrystals, and this increasing tendency of the d_33_ with the proportion of relative weight of DL-alanine can be seen as evidently demonstrating that the piezoelectric response in the d_33_ mode of the PVA/DL-alanine polycrystals originates from DL-alanine.

However, despite the higher weight ratio of DL-alanine, the PVA/DL-alanine polycrystal with an r of 3 was found to have the d_33_ of about 5 and ‒6 pC/N, smaller in magnitude than the d_33_ of the polycrystal with an r of 2, at the top and the bottom surfaces, and the reason for this is that a lot of DL-alanine was crystallized at the edge of the polycrystal where evaporation occurs quickly, so there was less DL-alanine, imparting piezoelectric properties, in the central part of the polycrystal from which the d_33_ piezoelectric coefficient is measured.^13^

### SEM images of the PVA/DL-alanine polycrystals

The d_33_ piezoelectric coefficient measurement indicated that the PVA/DL-alanine polycrystals have piezoelectricity in the d_33_ mode and also showed that the piezoelectric response of the PVA/DL-alanine polycrystals, together with the increasing trend of the d_33_ according to the relative weight ratio of DL-alanine to PVA, is attributed to the DL-alanine. However, further studies are required to understand how DL-alanine contributes to the development of the piezoelectricity of the PVA/DL-alanine polycrystals. In many studies, it has been reported that crystallized DL-alanine exhibits piezoelectricity.^15^^,^^16^^,^^25^ Therefore, the investigation of the process of DL-alanine crystallization in a solution mixture of PVA and DL-alanine can help in understanding the mechanism of piezoelectricity development of PVA/DL-alanine polycrystals. Accordingly, the cross-sectional crystal structures of the polycrystals were analyzed by SEM. The detailed information on the SEM analysis carried out in this study is presented in the STAR Methods section (see method details).

**Figure 2:**
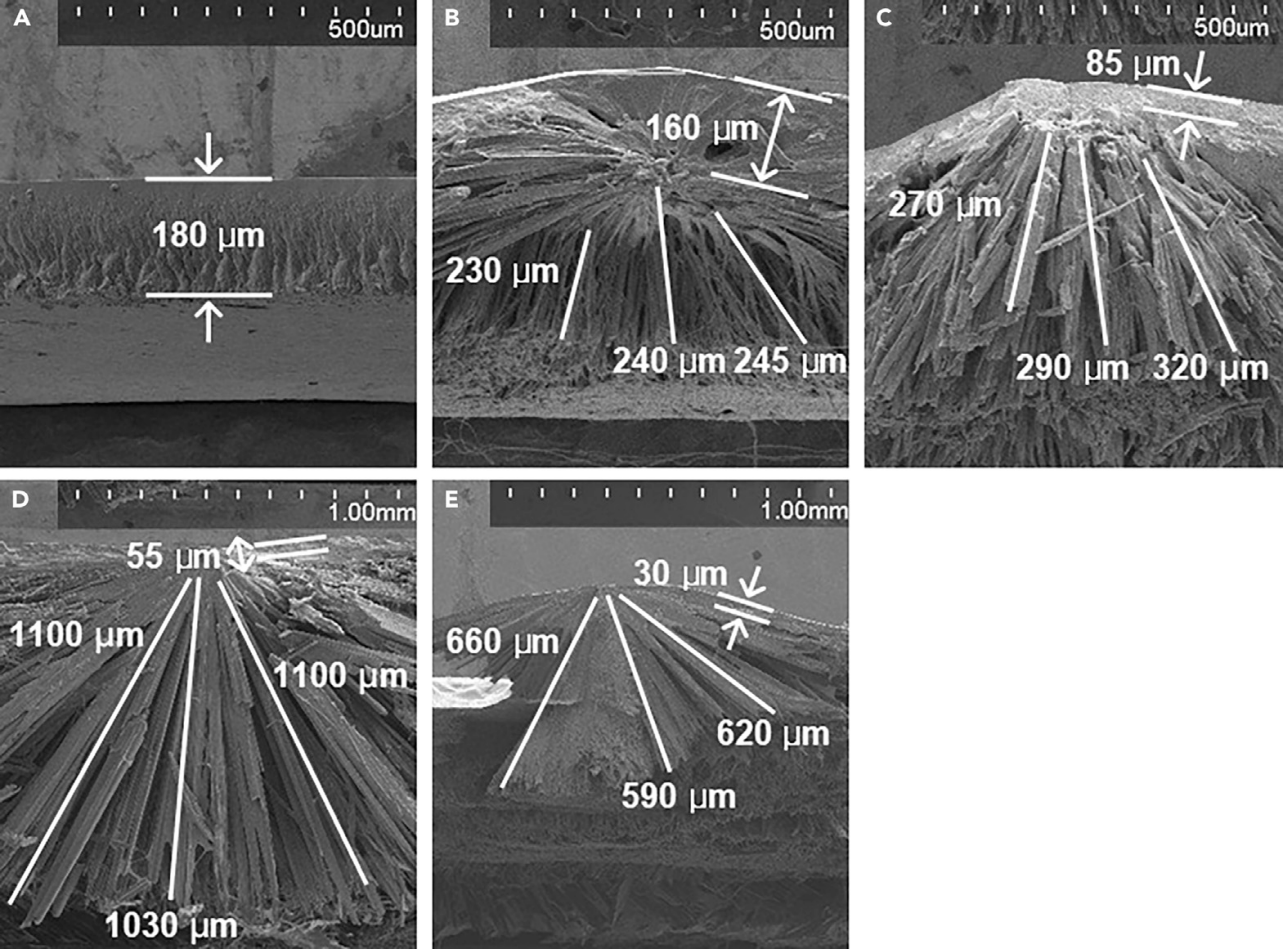
Cross-sectional SEM images of the PVA/DL-alanine polycrystals (A–E) Cross-sectional SEM images of the PVA/DL-alanine polycrystals with the r of (A) 0.2, (B) 0.5, (C) 1, (D) 2, and (E) 3. The scale bars are presented at the top of each figure. See also Figure S1.

In Figure 2A, the cross-sectional SEM image of the PVA/DL-alanine polycrystal with an r of 0.2 is shown. A slightly wrinkled morphology is observed at the lower part, but the overall crystal structure seems to be formed uniformly and evenly with a thickness of approximately 180 μm without noticeable structural features. However, as r increases, a bilayer structure begins to be observed in the cross-sectional crystal structures of the PVA/DL-alanine polycrystals (Figures 2B–2E), and the bilayer structure of the polycrystals can be divided into the upper and the lower parts.

The upper and the lower parts constituting the bilayer structure of the PVA/DL-alanine polycrystals appear to show completely different morphology, respectively. The morphology of the upper part is analogous to the morphology of the PVA/DL-alanine polycrystal with an r of 0.2. In other words, no distinct structural characteristics are observed. In contrast, the lower part shows a unique morphology that resembles a needle-like shape (see Figure S1 in the supplemental information section). The needle-like morphology observed in the lower part appears to be aligned nearly vertically or slightly obliquely. The needle-like morphology becomes apparent according to the weight ratio of DL-alanine constituting the PVA/DL-alanine polycrystals, so it can be confirmed that the length of the needle-like crystals increases from approximately 200 μm–1100 μm as r increases from 0.5 to 2.

It has been well known that the needle-like morphology is the representative morphology of crystallized DL-alanine amino acid.^25^^,^^26^^,^^27^^,^^28^^,^^29^ Therefore, the crystal structure of the PVA/DL-alanine polycrystals, displaying the needle-like morphology, can be seen as indicating that the lower part of the PVA/DL-alanine polycrystals are mainly comprised of the crystallized DL-alanine. Accordingly, it becomes evident that the increase in the length of the needle-like crystals is attributed to the increase of the weight ratio of DL-alanine constituting the polycrystals, and on the other hand, it is also suggested that the upper part, which shows no notable structural feature and becomes thinner from about 160 to 30 μm as the r increases from 0.5 to 3, predominantly consists of the PVA rather than DL-alanine.

In Figure 2E, it can be confirmed that the needle-like morphology of the polycrystal with an r of 3 is shorter than that of the polycrystal with an r of 2. This seems to be because of excessive crystallization of DL-alanine at the edge of the polycrystal owing to the considerable supersaturation, and given the decrease of the d_33_ observed at the polycrystal with an r of 3, it can be deduced that the immoderate weight ratio or concentration of DL-alanine in the solution would not be advantageous for the piezoelectricity of the PVA/DL-alanine polycrystals. As a result, the cross-sectional SEM analysis on the PVA/DL-alanine polycrystals suggests two facts about the piezoelectricity development of the PVA/DL-alanine polycrystals in the d_33_ mode. The first is that the d_33_ piezoelectricity development of the PVA/DL-alanine polycrystals is attributed to the DL-alanine crystallized in the needle-like morphology from the solution mixed with PVA, and the second is that the piezoelectric response of the polycrystals is significantly affected by the weight ratio of crystallized DL-alanine. In summary, the piezoelectric characteristics in the d_33_ mode of the PVA/DL-alanine polycrystals are determined by how DL-alanine is crystallized in the polycrystals, and thus, the XRD analysis was carried out to comprehend further the development of the piezoelectricity of the PVA/DL-alanine polycrystals in terms of the crystal structure.

### XRD patterns of the PVA/DL-alanine polycrystals

**Figure 3:**
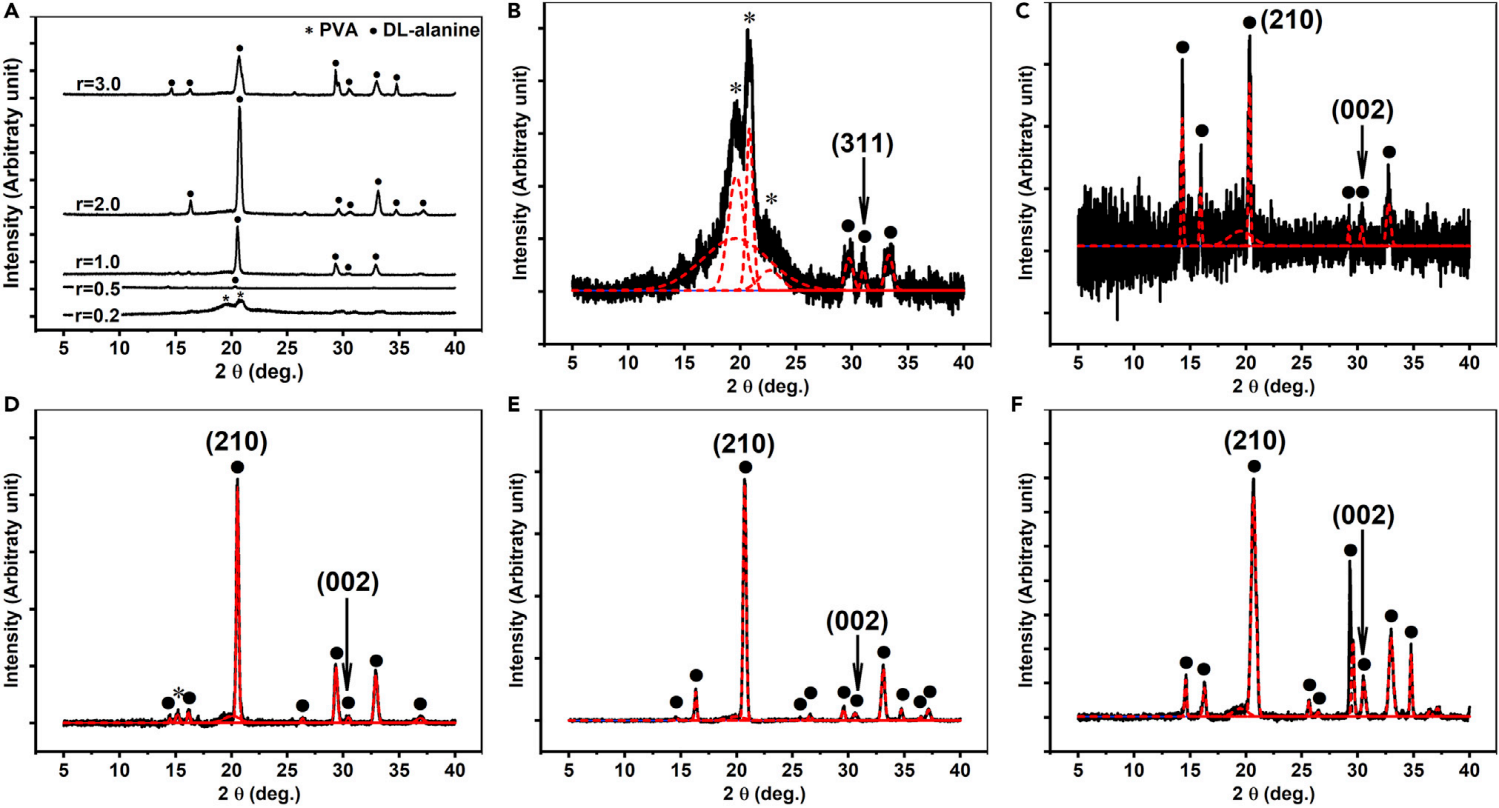
XRD patterns of the PVA/DL-alanine polycrystals (A–F) (A) Theta-two theta XRD patterns measured at the top surface of the PVA/DL-alanine polycrystals with the r of 0.2, 0.5, 1, 2, and 3 on the same scale. Deconvoluted XRD patterns of the PVA/DL-alanine polycrystals with the r of (B) 0.2, (C) 0.5, (D) 1, (E) 2, and (F) 3. Circles (•) and asterisks (∗) indicate whether the diffraction peaks originate from DL-alanine or PVA, respectively. See also Figures S2–S4, Tables S1 and S2.

The XRD analysis of the PVA/DL-alanine polycrystals was carried out in the theta (θ)–two theta (2 θ) scan mode, which is used to measure XRD patterns of materials that are not single-crystals. The XRD patterns of the polycrystals were measured at the top surface of them, i.e., the outer surface of the upper part, and the XRD patterns of all polycrystals are presented together in Figure 3A on the same scale, showing that there is a difference in the XRD patterns of the PVA/DL-alanine polycrystals.

In Figure 3B, the XRD pattern of the PVA/DL-alanine polycrystal with an r of 0.2 is shown. In the XRD pattern, two diffraction peaks, which seem to originate from the (10−1) and (101) faces of PVA, are observed at a 2 θ of approximately 19.7 and 20.9°.^30^^,^^31^^,^^32^^,^^33^^,^^34^^,^^35^ Several diffraction peaks corresponding to the faces of DL-alanine are also observed at 2 θ around and above 30°. However, the intensity of these peaks appears to be insignificant compared to the two peaks at 19.7 and 20.9°, which seems to be due to the fact that the polycrystal with an r of 0.2 is mainly composed of PVA rather than DL-alanine, and thus, the amount of DL-alanine crystallized therein is not much, given that the needle-like morphology of DL-alanine was rarely observed in the cross-sectional SEM image of the polycrystal in Figure 2A. Therefore, the absence of the piezoelectric characteristics of the PVA/DL-alanine polycrystal with an r of 0.2, which was revealed by its d_33_, can be understood to be due to the insufficient crystallization of DL-alanine according to its low concentration, and in the XRD pattern of the PVA/DL-alanine polycrystal with an r of 0.5, which is composed of more DL-alanine than this, a diffraction peak is observed at near 20.3° (Figure 3C).

The FWHM (full width at half maximum) of this diffraction peak was confirmed to be approximately 0.24°. For the two diffraction peaks of 19.7 and 20.9° of the polycrystal with an r of 0.2, their FWHM values were found to be 1.67 and 0.72°, respectively (see Table S1 in the supplemental information section). In other words, the FWHM of the peak at near 20.3° of polycrystal with an r of 0.5 is even smaller than the FWHM of the two peaks of the polycrystal with an r of 0.2, suggesting that the crystallite size of the crystal face that the peak at 20.3° represents is much larger than the crystallite size of the faces corresponding to the two main diffraction peaks of the polycrystal with an r of 0.2. Hence, given that the needle-like morphology, a typical crystal structure of DL-alanine, was verified in the cross-section of the polycrystal and the relative weight ratio of DL-alanine to PVA is higher in it than in the polycrystal with an r of 0.2, the diffraction peak at 20.3° observed in the XRD pattern of the polycrystal with an r of 0.5 can be seen as representing the (210) face of DL-alanine.^15^^,^^26^^,^^28^

DL-alanine, dissolved in solution, exists as zwitterions, that is, NH_3_^+^-CH(CH_3_)-COO^−^, with the amino groups (NH_3_^+^) and carboxylate groups (COO^−^) at its +c and −c ends, respectively. Therefore, DL-alanine crystals are formed by the repetitive hydrogen bonds between the amino groups (NH_3_^+^) and the carboxylate groups (COO^−^) at the c-axis ends, leading to the DL-alanine crystals displaying a needle-like morphology growing along its c-axis direction.^23^^,^^25^^,^^26^^,^^27^^,^^36^ As a result, the c-axis ends of the needle-like morphology, corresponding to the (001) and (00–1) faces, become polar faces because of the exposed amino and the carboxylate groups at the ends, and owing to the polar faces, DL-alanine can exhibit piezoelectricity along its c-axis according to the spontaneous polarization induced by the polar faces,^15^^,^^23^^,^^25^^,^^26^^,^^27^ and such spontaneous polarization formation according to the growth of crystals or nanostructures are observed in various organic and inorganic materials these days.^37^^,^^38^^,^^39^^,^^40^^,^^41^ Accordingly, for DL-alanine, a side face is inevitably formed along to the c-axis direction due to the growth in the needle-like morphology of DL-alanine, and this side face is the (210) face of the DL-alanine crystals. Therefore, the appearance of the diffraction peak corresponding to the (210) face of DL-alanine may indicate the c-axis-oriented growth of DL-alanine, and in this regard, the piezoelectricity in the d_33_ mode of the PVA/DL-alanine polycrystal with an r of 0.5 seems to originate from the formation of the needle-like morphology of DL-alanine according to its larger weight ratio of DL-alanine than that of the polycrystal with an r of 0.2.

As r increases, other diffraction peaks corresponding to the faces of the DL-alanine crystal begin to be observed in the XRD patterns, but the diffraction peak corresponding to the (210) face of DL-alanine is still dominant. This indicates that DL-alanine is better crystallized in the needle-like morphology with increasing r, which is in good agreement with the crystal structures observed in the cross-sectional SEM images of the PVA/DL-alanine polycrystals with a higher weight ratio of DL-alanine.

The change in the diffraction peak of the (210) face with respect to r seems to be analogous to that of the d_33_ piezoelectric coefficient of the PVA/DL-alanine polycrystals. In a study on the piezoelectric properties of DL-alanine crystals, it was suggested that the piezoelectric response of the DL-alanine crystals obtained by evaporating a supersaturated solution arises from the combined piezoelectric coefficients due to the randomly oriented DL-alanine crystals.^15^ However, for most DL-alanine crystals growing through solvent evaporation, the DL-alanine crystals with a needle-like morphology tended to grow parallel to the substrate, leading to their in-plane alignment on the substrate where the crystals grow. Accordingly, the face of DL-alanine crystals exposed most on the substrate on which they grow can be considered to be the (210) face; thus, most of the force applied to the DL-alanine polycrystals is transmitted to the (210) face. However, for the PVA/DL-alanine polycrystals, the face that receives the compressive force most is not the (210) face, but seems to be the faces at the ends of the needle-like morphology, considering the structure that the needle-like DL-alanine crystals are aligned vertically. Therefore, it seems not easy to explain the piezoelectricity of the PVA/DL-alanine polycrystals with the conventional piezoelectricity development mechanisms of DL-alanine suggested in other studies, so it is difficult to say that the change of the d_33_ of the polycrystals is attributed to the growth of the (210) face of DL-alanine in them.

In Figures 3B–3F, the XRD patterns of the PVA/DL-alanine polycrystals are deconvoluted. As shown in the figures, it can be confirmed that the XRD patterns are deconvoluted into many diffraction peaks of DL-alanine (see also Table S1 in the supplemental information section). Among these peaks, the diffraction peak corresponding to the (002) face of DL-alanine at about 30.6° is noteworthy, because the (002) face is substantially the same as the (001) face, the polar face of DL-alanine. In analyzing the crystal structure of nanostructured ZnO, the diffraction peak corresponding to the (002) face in the XRD pattern indicates that ZnO grows into a wurtzite structure, i.e., grows along the c-axis.^1^^,^^2^ From this standpoint, the appearance of the diffraction peak corresponding to the (002) face of DL-alanine can be considered to indicate that the DL-alanine crystals, which constitute the PVA/DL-alanine polycrystals, grow to be aligned in the out-of-plane direction, i.e., the 3-axis direction that is perpendicular to the top and bottom surfaces of the polycrystals.

Furthermore, the diffraction peak corresponding to the (201)/(011) faces, the polar +c end sometimes called (001), is observed only in the XRD patterns measured at the bottom surface of the PVA/DL-alanine polycrystals exhibiting the piezoelectricity (see Figures S2 and S3 and Table S2 in the supplemental information section),^28^ and the FT-IR spectra measured at the bottom surface of the polycrystals show only the absorbance bands derived from DL-alanine. On the other hand, the FT-IR spectra at the top surface show only the bands for PVA, not DL-alanine (see Figure S4 in the supplemental information section) so that it can be seen that the piezoelectric characteristics of the PVA/DL-alanine polycrystals in the d_33_ mode are due to the growth of DL-alanine being aligned in the 3-axis direction, i.e., the thickness direction of the bilayer structure of the PVA/DL-alanine polycrystals. Moreover, from the bottom surface XRD patterns of the PVA/DL-alanine polycrystals, it was discovered that the trend of the intensity of the diffraction peak of the (201)/(011) faces according to r of the polycrystals is very analogous to that of the d_33_ of them, which can be seen as strengthening the inference on the development of the piezoelectricity of the PVA/DL-alanine polycrystals, but additional study is needed to confirm this.

Here, the mechanism of the alignment of the needle-like morphology of the DL-alanine crystals along the 3-axis direction can be inferred. In the studies discussing the hydrogen bond formation between various functional groups, it was proposed that the hydroxyl groups (−OH) of PVA and the functional groups of amino acids, i.e., amino and carboxylate groups, can form hydrogen bonds.^13^^,^^42^^,^^43^ Therefore, likewise for PVA/DL-alanine polycrystals, the hydrogen bond formation between the hydroxyl groups of the PVA and the functional groups of the DL-alanine in the zwitterion form can be expected, and the nucleation of the DL-alanine seems to be initiated by the hydrogen bonds between the PVA and the zwitterionic DL-alanine along the PVA chain forming the upper part of the polycrystals, so DL-alanine can grow into the needle-like morphology below the top PVA layer, while forming the lower part of the PVA/DL-alanine polycrystals, as shown in the cross-sectional SEM images. In this respect, the reason that the PVA/DL-alanine polycrystal with an r of 3 can exhibit piezoelectric characteristics comparable to those of the polycrystal with an r of 2 in the d_33_ mode, despite the DL-alanine crystals being less grown in the needle-like morphology, seems to be attributed to the decent growth of the polar faces. The detailed information on the XRD and the FT-IR analysis performed on the PVA/DL-alanine polycrystals is presented in the STAR Methods section (see method details).

### Fabrication of the ZnO thin film piezoelectric device and the PVA/DL-alanine polycrystal-ZnO thin film heterostructured piezoelectric devices

**Figure 4:**
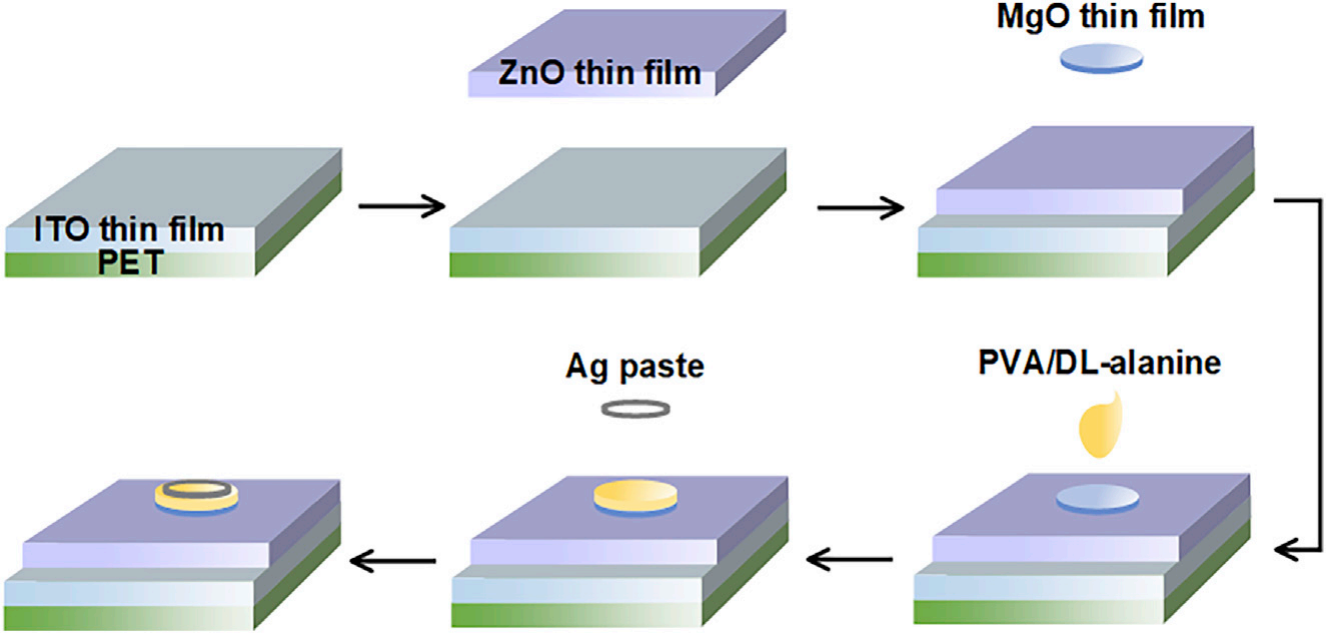
Fabrication of the ZnO thin film piezoelectric device and the PVA/DL-alanine polycrystal‒ZnO thin film heterostructured piezoelectric devices Schematic diagrams describing the fabrication of the ZnO thin film (S1) and PVA/DL-alanine polycrystal-ZnO thin-film heterostructured piezoelectric devices (S2–S6). See also Figure S5.

To confirm and evaluate the performance of the PVA/DL-alanine polycrystals as piezoelectric materials, six different piezoelectric devices (S1–S6) were fabricated. The fabrication process of the devices is presented in Figure 4. The piezoelectric device S1 is a device consisting of a single ZnO thin film layer deposited on an indium tin oxide (ITO)-coated polyethylene terephthalate (PET) substrate with a size of 2 × 3 cm^2^, and S2 to S6 are heterostructured devices in which a layer of PVA/DL-alanine polycrystal with an r of 0.2 (S2), 0.5 (S3), 1 (S4), 2 (S5), and 3 (S6) was formed on the ZnO thin film layer.

For devices S2 to S6, a MgO thin film was deposited on the ZnO thin film prior to forming the PVA/DL-alanine polycrystal layer to form identically patterned PVA/DL-alanine layers in all the heterostructured devices by exploiting the hydrophilicity of MgO. The deposition of ZnO thin film was conducted using a radio frequency (RF) magnetron sputtering system under a mixture of Ar and O_2_ gas ambient with a mass flow ratio of 4:1 at room temperature, and the deposition area of the ZnO thin film was adjusted to be 2 × 2.25 cm^2^ in all the devices. The thickness of the deposited ZnO thin film on an ITO-PET substrate was confirmed to be approximately 200 nm through the cross-sectional SEM image, and the piezoelectric property of the ZnO thin film was demonstrated by its grazing incidence XRD (GIXRD) pattern showing the wurtzite crystal structure (see Figure S5 in the supplemental information section).^1^^,^^2^

The MgO thin film, constituting the heterostructured piezoelectric devices (S2 to S6), was deposited to be approximately 20 nm in thickness in all the heterostructured devices using a shadow mask that has a circle-shape pattern with a radius of 0.35 cm through electron beam evaporation in vacuum at room temperature. The PVA/DL-alanine polycrystal layer was formed by dropping the solution of 0.05 mL where the PVA and DL-alanine were mixed with a specific weight ratio on the MgO thin film layer using a pipette. On the other hand, the Ag paste (P-100), which acted as an electrode, was formed in an area of about 0.16 cm^2^ in a ring shape using a shadow mask, because the edge of the formed PVA/DL-alanine polycrystal layer is more convex than its central part, leading to most of the force according to a periodic compression being delivered to this edge. The detailed information on the fabrication of the piezoelectric devices is provided in the STAR Methods section (see method details).

### Piezoelectric performance of the ZnO thin film piezoelectric device and the PVA/DL-alanine polycrystal-ZnO thin film heterostructured piezoelectric devices

To measure the open-circuit voltages of S1–S6 in the d_33_ mode, all the piezoelectric devices were subjected to a periodic compression test. The periodic compression was implemented using a linear motor equipped with a cylinder-shaped pusher made of polylactic acid whose effective area in contact with the devices is approximately 0.4 cm^2^, and the force applied to the devices was adjusted by controlling the frequency of the periodic compression, as presented in Figure S6 in the supplemental information section.

**Figure 5:**
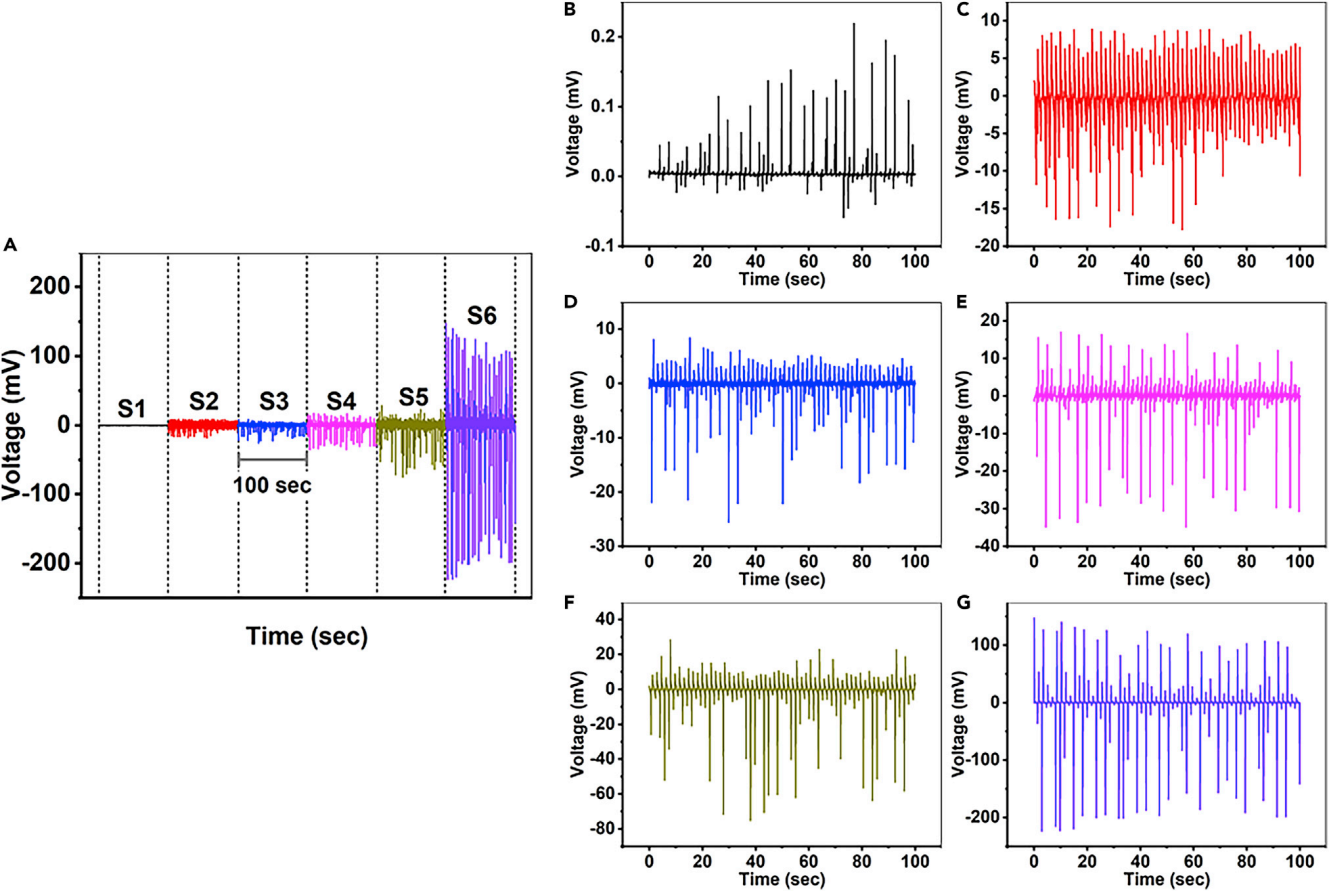
Piezoelectric open-circuit voltages of the ZnO thin film piezoelectric device and the PVA/DL-alanine polycrystal-ZnO thin film heterostructured piezoelectric devices in the d_33_ mode (A–G) Piezoelectric open-circuit voltages of the ZnO thin film piezoelectric device (S1) and the PVA/DL-alanine polycrystal/ZnO thin film heterostructured piezoelectric devices (S2–S6) in the d_33_ mode (A) on the same scale, and the individual plotting of the open-circuit voltages of (B) S1, (C) S2, (D) S3, (E) S4, (F) S5, and (G) S6 when the compression frequency is 0.77 Hz. The time scale bar of 100 sec is presented in (A). See also Figures S6–S9.

The piezoelectric open-circuit voltages of S1–S6 according to the periodic compression of 0.77 Hz are shown in Figure 5A. In the figure, it is noteworthy that the piezoelectric output of device S1, composed of a single ZnO piezoelectric thin film, is much smaller than that of the others. The piezoelectric voltage of device S1 is plotted individually in Figure 5B, wherein it can be confirmed that the device S1 generates the piezoelectric output periodically according to the compression, but the peak-to-peak value of the output voltage is approximately less than 0.2 mV. The open-circuit voltage of device S2 is shown in detail in Figure 5C. As in the case of device S1, device S2 was also confirmed to be able to generate the piezoelectric output according to the periodic compression. The peak-to-peak value of the generated voltage of device S2 is approximately 20 mV, which is much larger than that of device S1. However, the output voltage of device S2 is inferred to be mainly attributed to the reduced screening effect owing to the dielectric properties of the PVA/DL-alanine polycrystal layer rather than the piezoelectricity of PVA/DL-alanine, because DL-alanine crystallized in the needle-like morphology was rarely found in the polycrystal layer, like in the free-standing PVA/DL-alanine polycrystal with an r of 0.2 in Figure 2.

As shown in Figures 5D–5G, the open-circuit voltages of the heterostructured piezoelectric devices appear to be enhanced with an increase in the weight ratio of DL-alanine. In particular, a distinct improvement in the piezoelectric characteristics is confirmed in the open-circuit voltage of device S6 whose peak-to-peak value is about 300 mV, which is more than 100 times greater than 0.2 mV, the piezoelectric output of device S1. This enhancement in the piezoelectric output of device S6 can be inferred to be attributed to the significantly reduced crystallization of DL-alanine at the edge, which does not contribute to the development of piezoelectricity, unlike the case of the free-standing film, and meanwhile, the thickness ratio between the upper part mainly composed of PVA and the lower part consisting of DL-alanine crystals, determining the piezoelectric properties of materials according to the trade-off relationship between the screening effect and the dielectric loss by the dielectric layer constituting piezoelectric materials, also can be considered to contribute to the enhancement of the piezoelectric output of S6,^1^ and thus, it seems to be for this reason that the trends of the d_33_ and the piezoelectric open-circuit voltages of the devices presented in Figure 5 according to the relative weight ratio of DL-alanine in the polycrystals are different. Consequently, the reliable and significant d_33_ piezoelectric characteristics of the PVA/DL-alanine polycrystals have been demonstrated through a comparison of the piezoelectric voltages of the devices with a single layer of ZnO thin film and the heterostructured devices composed of the PVA/DL-alanine polycrystal and ZnO thin film, and in Figures S7–S9 in the supplemental information section, the piezoelectric open-circuit voltages according to the periodic compression of the free-standing PVA/DL-alanine polycrystals are presented, in which the tendency of the piezoelectric open-circuit voltages in proportion to the applied force can be confirmed (the detailed information on the measurement of the piezoelectric open-circuit voltages of the free-standing PVA/DL-alanine polycrystals is provided in the STAR Methods section (see method details)). On the other hand, the piezoelectric output voltage generated by the devices seems small, considering the d_33_ of the PVA/DL-alanine polycrystals. This appears to be due to the dielectric loss caused by the dielectric constant mismatch between the DL-alanine and PVA. Therefore, in the near future, we expect that a piezoelectric device with enhanced performance will be developed if a polymer having a dielectric constant similar to that of DL-alanine is used.

## Discussion

In this study, the fabrication of piezoelectric materials wherein PVA polymer and DL-alanine amino acids are combined, i.e., PVA/DL-alanine polycrystals, has been proposed, and their piezoelectric characteristics were investigated. The piezoelectric properties of the PVA/DL-alanine polycrystals were evaluated by measuring their d_33_ piezoelectric coefficients, and it was confirmed that the d_33_ piezoelectric properties of the polycrystals are improved as the weight ratio of DL-alanine constituting them increases. Among the fabricated PVA/DL-alanine polycrystals, the polycrystal composed of PVA and DL-alanine with a weight ratio of 1:3 exhibited a d_33_ of about 5 pC/N on average which is nearly the same as that of the longitudinal piezoelectric coefficient of DL-alanine single crystal. To reveal the mechanism of the piezoelectric development of the polycrystals, the crystal structure of the PVA/DL-alanine polycrystals was investigated through their cross-sectional SEM images, XRD patterns, and FT-IR spectra. The analysis on the crystal structure of the PVA/DL-alanine polycrystals suggested that the PVA/DL-alanine polycrystals have a bilayer structure composed of the upper and the lower part, composed of mainly PVA and DL-alanine, respectively, and it was indicated that the DL-alanine crystals of needle-like morphology aligned to the 3-axis direction, forming the lower part, are the origin of the piezoelectric characteristics of the PVA/DL-alanine polycrystals. This is a basic study to promote the design and fabrication of piezoelectric materials using DL-alanine amino acids. Therefore, further studies are essential to develop practical piezoelectric devices composed of DL-alanine-based piezoelectric materials. We hope that this study will serve as a cornerstone for further research on excellent amino acid-based piezoelectric materials with biodegradable and biocompatible properties, as well as DL-alanine-based piezoelectric materials.

### Limitations of study

The piezoelectric characteristics of the PVA/DL-alanine polycrystals in the d_33_ mode were confirmed experimentally by measuring the piezoelectric open-circuit voltages of the PVA/DL-alanine polycrystal-ZnO thin film heterostructured piezoelectric devices. It was also discussed that the piezoelectricity of the PVA/DL-alanine polycrystals is attributed to the alignment of the DL-alanine crystals to the thickness direction, i.e., the 3-axis direction, by the hydrogen bond formation between the functional groups of the PVA and DL-alanine, but in-depth study accompanied with additional experiments on the piezoelectricity in the d_33_ mode of DL-alanine-based materials combined with other polymers seems required to demonstrate the suggestion on the piezoelectricity development mechanism of the PVA/DL-alanine polycrystals.

## STAR★Methods

### Key resources table

**Table:** 

REAGENT or RESOURCE	SOURCE	IDENTIFIER
**Chemicals, peptides, and recombinant proteins**		

Polyvinyl alcohol	Acros Organics	CAS# 9002-89-5
DL-alanine	Alfa Aesar	CAS# 302-72-7
Acetone	OCI company Ltd.	CAS# 67-64-1
Ethanol	OCI company Ltd.	CAS# 64-17-5
Zinc oxide (ZnO) material sputtering target	iTASCO	CAS# 1314-13-2
Magnesium oxide (MgO) material electron beam evaporation source	iTASCO	CAS# 1309-48-4
P-100 conductive Ag paste	CANS	N/A

**Software and algorithms**		

Origin 2019	Origin Lab	https://originlab.com/
PDXL	Rigaku	https://www.rigaku.com/

**Other**		

ITO-coated PET	Hanalintech	N/A
Pipette	LABSMRO	http://www.labsmro.com/
YE2730A d_33_ meter	Sinocera	http://www.sinocera.net/
SMC 12R-plus sputter coater	Semian	https://www.semian.co.kr/
S-4800 FE-SEM system	Hitachi	https://www.hitachi.com/
Ultima IV x-ray diffractometer	Rigaku	https://www.rigaku.com/
Nicolet iS50 FT-IR spectrometer	Thermo Fisher	https://www.thermofisher.com/
WUC ultrasonic cleaner	DAIHAN	http://www.labsmro.com/
HVS-340 magnetron sputtering system	Hanvac	http://hanvac.kr.ecplaza.net/
Polyimide (PI) tape	AS ONE	https://www.as-1.co.jp/
Conductive aluminum (Al) tape	Haesung	N/A
Conductive copper (Cu) tape	3M	https://www.3m.com/

### Resource availability

#### Lead contact

Further information and requests for resources and reagents should be directed to and will be fulfilled by the lead contact, Giwan Yoon (gwyoon@kaist.ac.kr).

#### Materials availability

This study did not generate new unique reagents.

### Method details

#### Fabrication of PVA/DL-alanine polycrystals

PVA/DL-alanine polycrystals were obtained by evaporating solutions in which 10% weight per volume (w/v) solutions of polyvinyl alcohol (PVA) and DL-alanine were mixed. The PVA solution of 10% w/v was prepared by dissolving PVA granules (87–89% hydrolysis; molecular weight of 13,000–23,000; Acros Organics, Belgium) in deionized water and continuously stirring for 2 h at 353 K, and then, it was cooled to room temperature (∼293 K). Similarly, the DL-alanine solution of 10% w/v was prepared by dissolving DL-alanine powder (99% purity, Alfa Aesar, USA) in deionized water using a magnetic stirrer for 2 h; however, unlike the PVA solution, the dissolution of DL-alanine was performed at room temperature.

Both the prepared PVA and DL-alanine solutions were clear without impurities. The respective solutions were aliquoted into vials with specific volume ratios and stirred constantly at room temperature to adjust the weight ratio of the PVA and DL-alanine (PVA to DL-alanine) constituting the polycrystals to be 1:0.2, 1:0.5, 1:1, 1:2, and 1:3. In order to obtain the PVA/DL-alanine polycrystals, the solutions where the DL-alanine and PVA solutions are mixed in the specific weight ratios were aliquoted into a circle-shaped silicon mold with a radius of about 3 cm, and the volume of the solution was adjusted to be about 12 mL for all polycrystals in order to fill the mold without an empty area, and the aliquoted solutions were evaporated in a dry oven at 333 K for 4 h. The crystallization of the PVA/DL-alanine solution was found to commence at its edge and then propagated to the center, where PVA/DL-alanine polycrystals formed more densely at the edge than at the center.

After the solutions were completely evaporated in a dry oven at 333 K, the PVA/DL-alanine polycrystals self-formed in the molds were removed from the oven and cooled to room temperature prior to peeling them off, and all PVA/DL-alanine polycrystals were found to be flat and have uniform surfaces everywhere except at the edge where they were bent upwards owing to the meniscus by capillary action. Furthermore, they exhibited better durability for vertical pressing or scratching than pure DL-alanine crystals obtained by evaporating the DL-alanine-dissolved solution. The PVA/DL-alanine polycrystals for measuring the d_33_ piezoelectric coefficient were used without cutting separately, and those required for the cross-sectional SEM, XRD, and FT-IR analysis were prepared by cutting them into a square shape of 2 × 2 cm^2^.

#### Measurement of d_33_ piezoelectric coefficient of the PVA/DL-alanine polycrystals

The d_33_ piezoelectric coefficient was measured for the as-formed, uncut PVA/DL-alanine polycrystals. The d_33_ piezoelectric coefficient was measured under a quasi-static force, and the magnitude of the force applied to the polycrystals was 0.25 N at a frequency of 110 Hz by using a commercial d_33_ meter (YE2730A, Sinocera, China), and in order to confirm that the PVA/DL-alanine polycrystals have effective piezoelectric characteristics in the d_33_ mode, the d_33_ piezoelectric coefficient was measured at ten different sites on the top and bottom surfaces of each polycrystal. The average and standard deviation of the d_33_ were calculated from the measured d_33_ piezoelectric coefficients. All d_33_ piezoelectric coefficient measurements were performed in air at room temperature, ∼291 K, and the temperature was automatically controlled by an air conditioner.

#### Cross-sectional SEM analysis of the PVA/DL-alanine polycrystals

PVA/DL-alanine polycrystal samples of 2 × 2 cm^2^ size were frozen in liquid nitrogen for approximately 5 s and then cut to a suitable size to obtain clean cross-sectional SEM images of the PVA/DL-alanine polycrystals. The cut samples were fixed to the SEM sample holder so that their cross-sections were exposed for photography on the SEM holder, then the cut samples were coated with platinum (Pt) thin film through DC sputtering in an Ar gas ambient at a power of about 20 mA × 500 V for about 4–5 min by employing a sputter coater (SMC 12R-plus, Semian, South Korea) in vacuum of 5 × 10^−2^ mbar to obtain better cross-sectional SEM images by improving the electrical conductivity of the cross sections in which the SEM images are taken. The cross-sectional SEM images of PVA/DL-alanine polycrystals were taken by S-4800 FE-SEM system (Hitachi, Japan), and the voltage and current were set to 10 kV and 10 μA, respectively, and the measurement was carried out in vacuum at room temperature without intentional cooling. See also Figures S1 and S10 in the supplemental information section, and in Figure S10, the change of the thickness of the upper and the lower parts of the PVA/DL-alanine polycrystals according to the r is presented.

#### XRD analysis of the PVA/DL-alanine polycrystals

For the XRD analysis, the PVA/DL-alanine polycrystal samples of 2 × 2 cm^2^ size were used. The XRD patterns of the samples were measured by the theta-two theta scan mode by employing Ultima IV x-ray diffractometer (RIGAKU, Japan). The theta–two theta XRD patterns of the PVA/DL-alanine polycrystals were measured from 5 to 40°, and the sampling rate was set to 4°/min with a sampling step of 0.01°. Cu k-alpha (i.e, both Cu k-alpha 1 and Cu k-alpha 2) x-rays were used, and before the measurement, the beam alignment process was always performed. A divergence height limit (DHL) slit of 5 mm was used, and all samples were analyzed without being attached to a separate substrate. The diffraction peaks of the XRD patterns were validated using the PDXL XRD analysis software (Rigaku, Japan). XRD patterns of the PVA/DL-alanine polycrystals were measured at both the top and the bottom surfaces of them, and all the measurement was carried out in air at room temperature. The deconvolution of the measured XRD patterns of the PVA/DL-alanine polycrystals was conducted by using Origin 2019. See also Figures S2 and S3, Tables S1 and S2.

#### FT-IR analysis of the PVA/DL-alanine polycrystals

PVA/DL-alanine polycrystal sample of 2 × 2 cm^2^ size was used in the FT-IR analysis, too. There was no pre-treatment prior to the FT-IR analysis likewise the XRD analysis. Nicolet iS50 FT-IR spectrometer (Thermo Fisher, USA) was employed to obtain the FT-IR spectra of the polycrystals, and the measurement was performed in air at room temperature. The analysis range was set from 400 to 4000 cm^−1^, and the FT-IR spectra of the PVA/DL-alanine polycrystals were measured at both the top and the bottom surfaces of the polycrystals. See also Figure S4.

#### Fabrication of the ZnO thin film piezoelectric device and the PVA/DL-alanine polycrystal‒ZnO thin film heterostructured piezoelectric devices

Prior to depositing the MgO and ZnO thin films, the ITO-coated PET substrates (Hanalintech, South Korea) were cut into a rectangular shape with a size of 2 × 3 cm^2^. Then, the cut ITO-coated PET substrates were cleaned with acetone (OCI company Ltd., South Korea) and ethanol (OCI company Ltd., South Korea) consecutively in an ultrasonic cleaner (WUC, DAIHAN) for 5 min, respectively, at room temperature, and after this, they were washed with deionized water and dried under a halogen lamp for enough time to remove the humidity that could remain on the substrates.

The deposition of the ZnO thin film was performed by using a RF magnetron sputtering system (HVS-340, Hanvac, South Korea). Before depositing the ZnO thin film, pre-sputtering to remove the contaminants that could exist on the ZnO sputtering target (iTASCO, South Korea) was conducted for 10 min at in Ar gas ambient at 10 mtorr, and after the pre-sputtering was done, the deposition of the ZnO thin film was performed immediately by opening the closed shutter which is used to prevent the sputtering while closed. The RF power used to deposit the ZnO thin film was maintained to be 160 W, and the vacuum was fixed to be 10 mtorr during the deposition. The sputtering gas was Ar. O_2_ gas was introduced together with Ar, which is to compensate the oxygen deficiency in the ZnO thin film. The flow rate ratio of Ar to O_2_ was set to 4 to 1, and the deposition was performed for 30 min at room temperature without heating. See also Figure S5.

For the PVA/DL-alanine polycrystal-ZnO thin film heterostructured piezoelectric devices, MgO thin film was deposited directly over the ZnO thin film. The deposition of the MgO thin film was carried out by employing an electron beam evaporation technique at room temperature, and like the ZnO thin film deposition, the MgO source (iTASCO, South Korea) was pre-evaporated to remove contaminants present on the source material in advance for about 5 min prior to starting the deposition of the MgO thin film. The voltage and current to evaporate the MgO source were set to 7 kV and about 10 mA, and the thickness of the MgO thin film was measured in real-time through a thin-film thickness monitor built in the electron beam evaporator. The evaporation of the MgO was conducted at room temperature without additional heating, and the vacuum was maintained at about 10^−6^ torr during the deposition. For MgO thin film, it was deposited in a circular shape with a radius of 0.35 cm by using a shadow mask composed of polylactic acid.

The PVA/DL-alanine polycrystal layer for the heterostructured devices was formed by dropping the PVA-DL-alanine-mixed solution of 0.05 mL on the MgO thin film using a pipette. Owing to the hydrophilicity of the MgO, the solution naturally spread evenly along the circular pattern of the MgO thin film, and thus, the PVA/DL-alanine polycrystal layer formed the identical circular pattern. The top electrode of the piezoelectric energy harvesters was formed by conductive Ag paste (P-100, CANS). The Ag electrode was made in a ring-shaped pattern of with a size of 0.16 cm^2^ by using a shadow mask, and this is because the edge of the PVA/DL-alanine polycrystal layer was formed in a convex shape upward, while the middle part of the layer was relatively flat, and thus, it was expected that the piezoelectric characteristics of the polycrystals occur the most at the edge when the piezoelectric devices were stimulated under the d_33_ mode.

#### Measurement of the piezoelectric open-circuit voltages of the free-standing PVA/DL-alanine polycrystals

Due to the limitations of existing measuring equipment, used in the measurement of the ZnO thin film and the PVA/DL-alanine polycrystal‒ZnO thin film heterostructured piezoelectric devices, for the size and shape of the devices or materials to be measured, the piezoelectric open-circuit voltage measurement of the free-standing polycrystals was performed with equipment different from the used in measuring of the ZnO thin film and the PVA/DL-alanine polycrystal‒ZnO thin film heterostructured piezoelectric devices. Accordingly, the polycrystals with a circle shape whose radius is 2 cm were prepared separately, and only the polycrystals whose r is 1, 2, and 3 were fabricated and measured. This is because, in the case of the polycrystals with an r of 0.2 and 0.5, they were distorted in a circle shape with a radius of 2 cm and were not formed flat, making it difficult to measure ordinarily. In order to prevent the triboelectric effect that could occur by the periodic compression and release between the polycrystals and the compressor, triboelectric effect prevention layer (tribo-prevention layer), composed of electrically insulating polyimide (PI) tape (thickness: 55 μm, AS ONE, Japan), conductive aluminum (Al) (Heasung, South Korea) and copper (Cu) (thickness: 60 μm, 3M, USA) tapes, was formed on the polycrystals,^44^ and the effect of the tribo-prevention layer on the reduction of the triboelectric effect was confirmed by measuring and comparing the triboelectric effect occurring between a PVA film and the polyurethane compressor, suggesting that the triboelectric effect, which could occur from the periodic compression and release between the polycrystals and the compressor, is significantly reduced, so the voltages measured from the PVA/DL-alanine polycrystals is caused by the piezoelectric properties of the PVA/DL-alanine polycrystals (see Figures S7–S9 in the supplemental information section). The contact area between the compressor made of hexahedral polyurethane and the polycrystals was 1.3 × 1.3 cm^2^, and the moving speed of the compressor was set to 50 mm/s in all measurements. There was a delay of 0.5 s between a cycle of the periodic compression and release.

## Data Availability

• All data reported in this paper will be shared by the lead contact upon reasonable request. • This study does not report original codes. • Any additional information required to reanalyze the data reported in this paper is available from the lead contact upon request.
