# Supplementary material for: Piezoelectric characteristics of PVA/DL-alanine polycrystals in d33 mode
Source: iScience. 2022 Dec 8;26(1):105768. doi: 10.1016/j.isci.2022.105768 (PMC9800289; doi:10.1016/j.isci.2022.105768)
Supplement: Document S1. Figures S1–S10 and Tables S1 and S2 [file mmc1.pdf]

iScience, Volume *26*

Supplemental information

Piezoelectric characteristics of PVA/DL-alanine polycrystals in d_{33} mode

Buil Jeon, Dongsoo Han, and Giwan Yoon

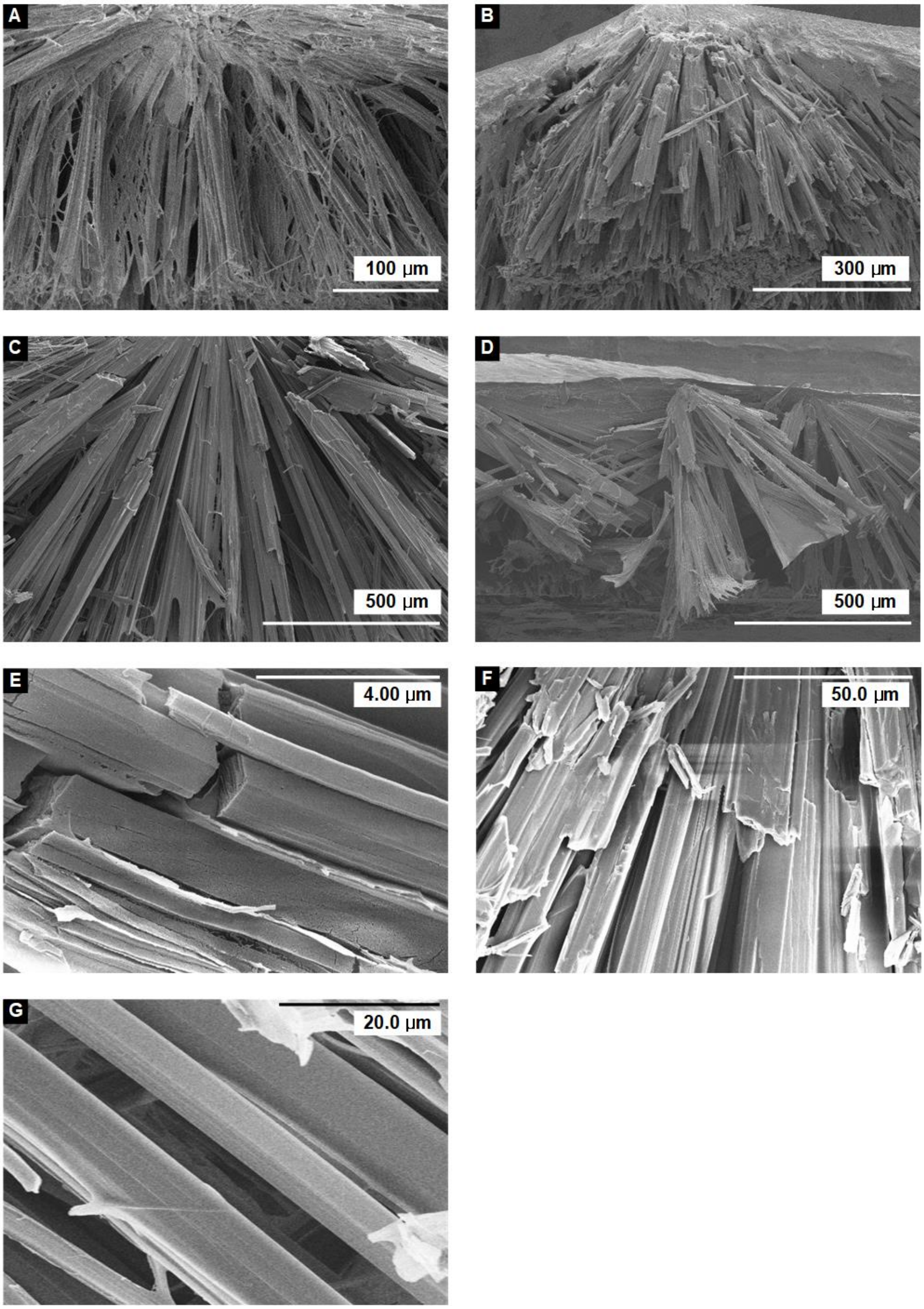

Figure S1. Enlarged cross-sectional SEM images of the PVA/DL-alanine polycrystals, related to Figure 2. Enlarged cross-sectional SEM images of the PVA/DL-alanine polycrystals with an r of (A) 0.5, (B) 1, (C) 2, and (D) 3. (E-G) SEM images of the needle-like morphology observed in the PVA/DL-alanine polycrystal with an r of 2. The scale bars are presented at the top and the bottom of each figure.

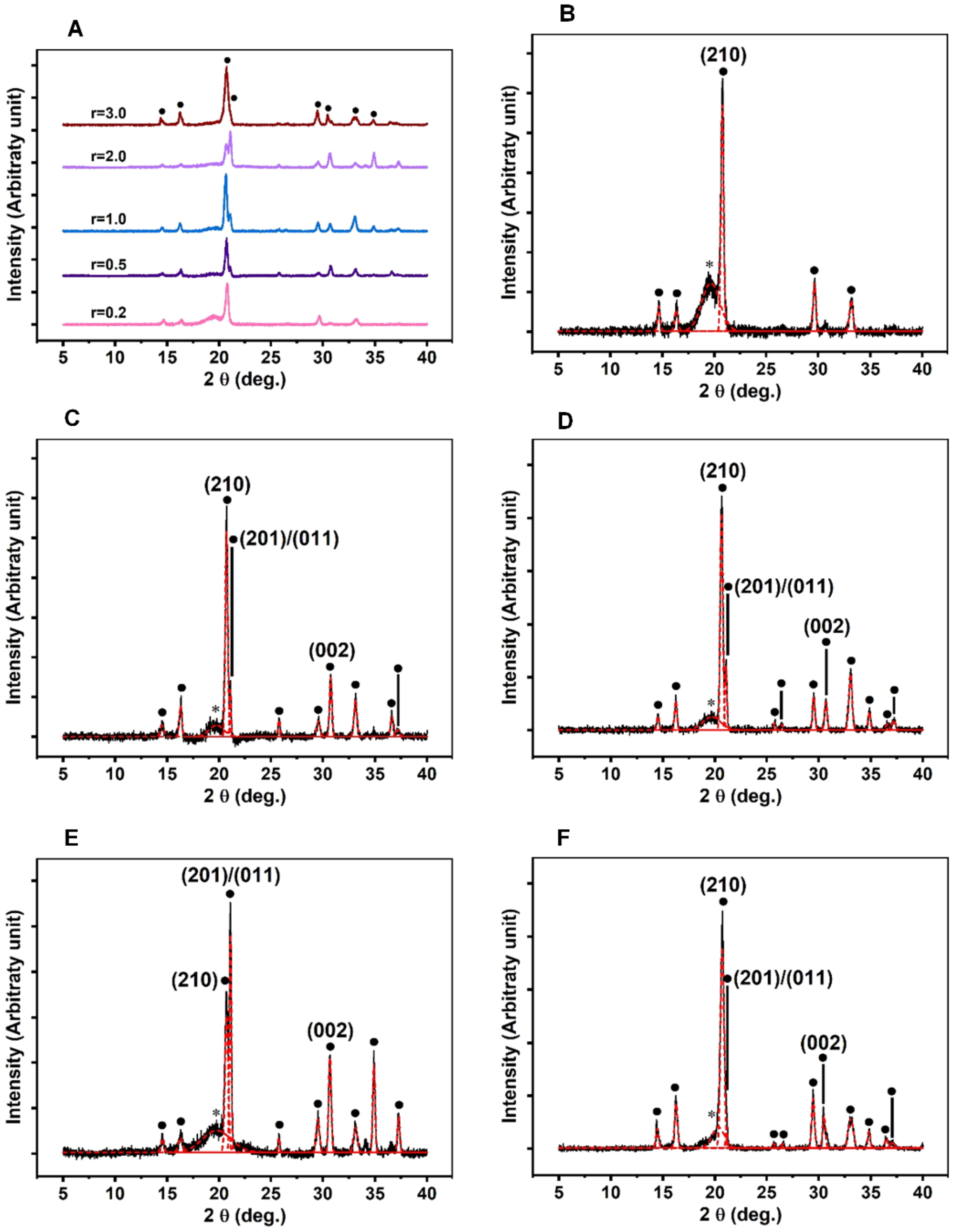

Figure S2. Theta-two theta XRD patterns measured at the bottom surface of the PVA/DL-alanine polycrystals, related to Figure 3 and STAR Methods. (A) XRD patterns measured at the bottom surface of the PVA/DL-alanine polycrystals with different r. Individual XRD patterns of the PVA/DL-alanine polycrystals with an r of (B) 0.2, (C) 0.5, (D) 1, (E) 2, and (F) 3 with deconvolution. Circles (•) and asterisks (∗) indicate whether the diffraction peaks originate from DL-alanine or PVA, respectively, and the face corresponding to the diffraction peak is labeled.

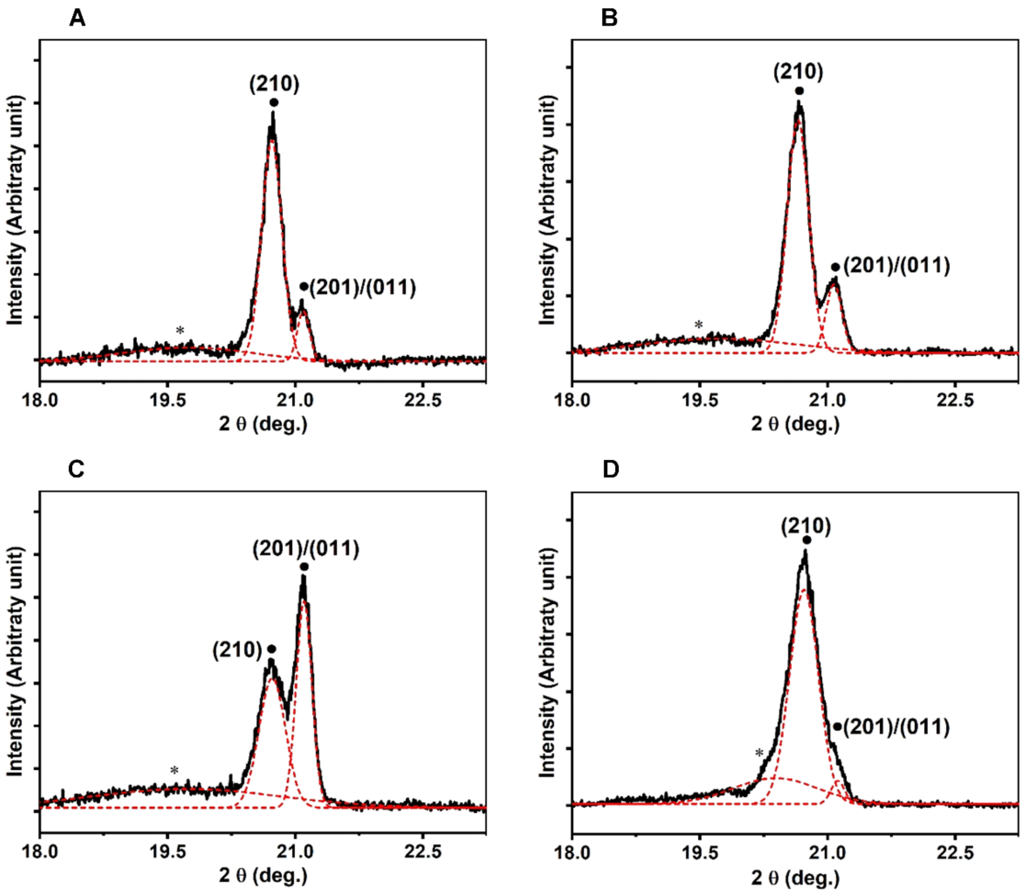

Figure S3. Enlarged theta-two theta XRD patterns measured at the bottom surface of the PVA/DL-alanine polycrystals, related to Figure 3 and STAR Methods. Enlarged XRD patterns of the PVA/DL-alanine polycrystal with an r of (A) 0.5, (B) 1, (C) 2, and (D) 3.

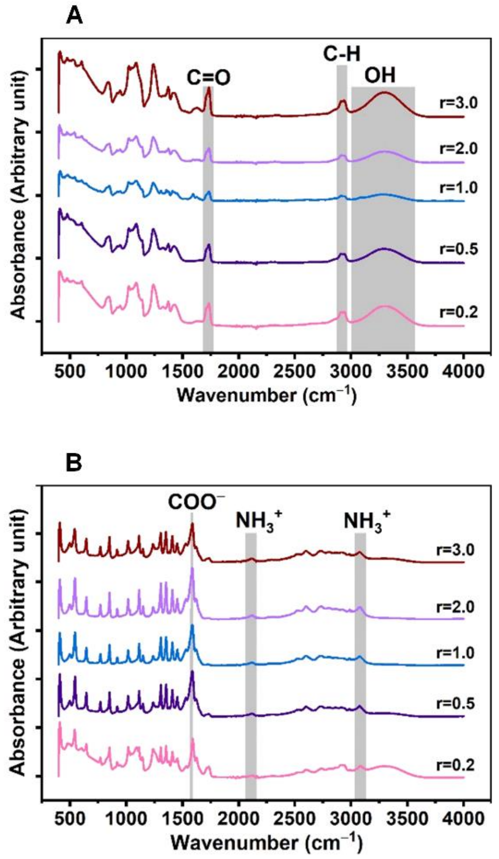

Figure S4. FT-IR spectra of the PVA/DL-alanine polycrystals, related to Figure 3 and STAR Methods. FT-IR spectra measured at the (A) top surface and the (B) bottom surface of the PVA/DL-alanine polycrystals with different r.

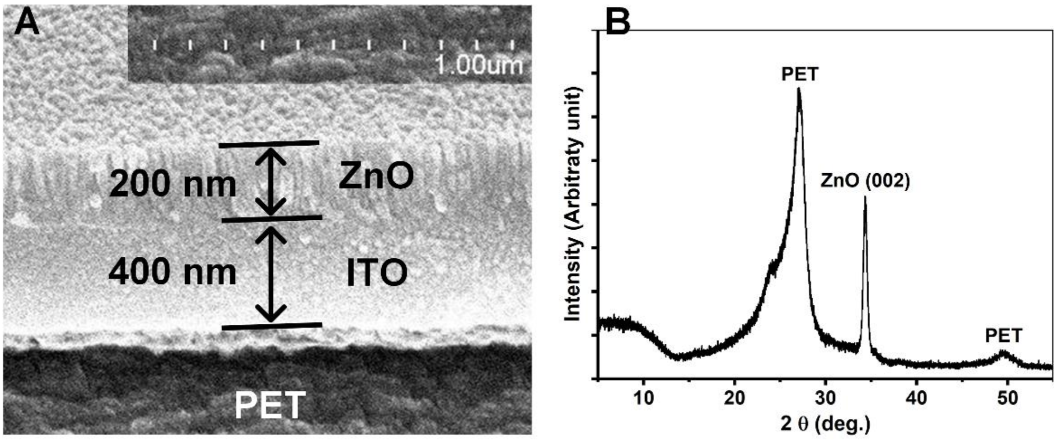

Figure S5. Cross-sectional SEM image and GIXRD pattern of the ZnO thin film deposited on an ITO-coated PET substrate, related to Figure 4. (A) Cross-sectional SEM image and (B) GIXRD pattern of the ZnO thin film deposited on an ITO-coated PET. The appearance at about 34.8 ° of the diffraction peak corresponding to the (002) face of ZnO can be considered to indicate that the ZnO is in wurtzite structure that is piezoelectric. The scale bar is presented at the top of (A).

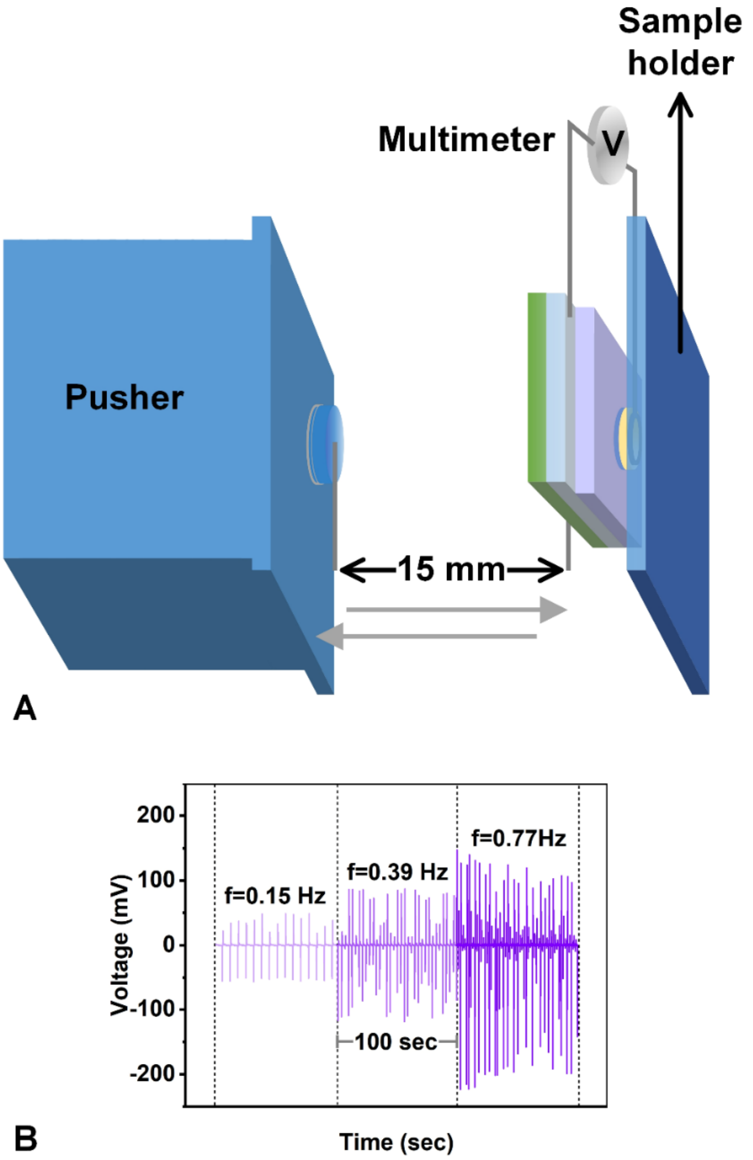

Figure S6. Periodic compression to the piezoelectric devices and piezoelectric open-circuit voltage of S6 device according to different compression frequency, related to Figure 5. (A) Schematic diagram describing the linear motor system used to implement the d_{33} mode through a periodic compression. (B) Piezoelectric open-circuit voltage of S6 device according to three different compression frequencies. The weight of the linear motor including the cylinder-shaped pusher is about 0.25 kg. The sample holder used to hold the piezoelectric devices tightly was made of polylactic acid plastic. The frequency of the linear motor employed to measure the open-circuit voltage presented in Figure 5 was 0.77 Hz, and as shown in Fig. S6B, the frequency was able to be controlled to be about 0.15 and 0.39 Hz by a lab-made linear motor controller. The distance where the repetitive linear movement of the motor was done was fixed to be 15 mm in all the measurements. The schematic diagram describing the linear motor system used to implement the d_{33} mode through a periodic compression is presented in Fig. S6A. On the other hand, the piezoelectric open-circuit voltage of S6 device according to three different compression frequencies are shown in Fig. S6B, where it can be confirmed that the magnitude of the open-circuit voltage increases according to the increase of the frequency. This suggests that the piezoelectric devices composed of the PVA/DL-alanine polycrystal exhibit better piezoelectric performance in a high-speed operation. Here, as the frequency increases, the reason why the waveform of the open-circuit voltage becomes more uneven seems to be due to that the measurement speed of the multimeter is not fast enough to measure the open-circuit voltage. The time scale bar of 100 sec is presented in (B).

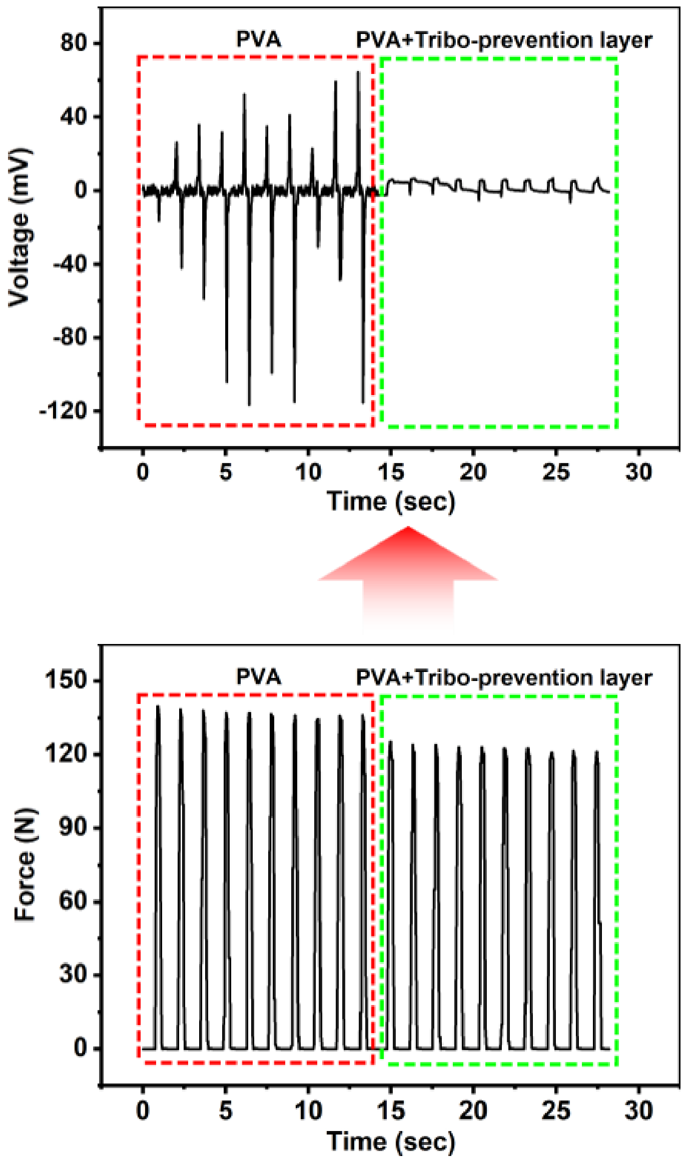

Figure S7. Effect of tribo-prevention layer that is formed in a PVA film, showing the reduction of the triboelectric effect, related to Figure 5 and STAR Methods. (Upper) The voltage in a red-dashed box is the triboelectric output between PVA and polyurethane compressor. The voltage in a green-dashed box shows the reduction of the triboelectric effect between the PVA and the compressor, demonstrating that the tribo-prevention layer can reduce the triboelectric effect. (Lower) The force of about 120 N applied to the PVA film [S1].

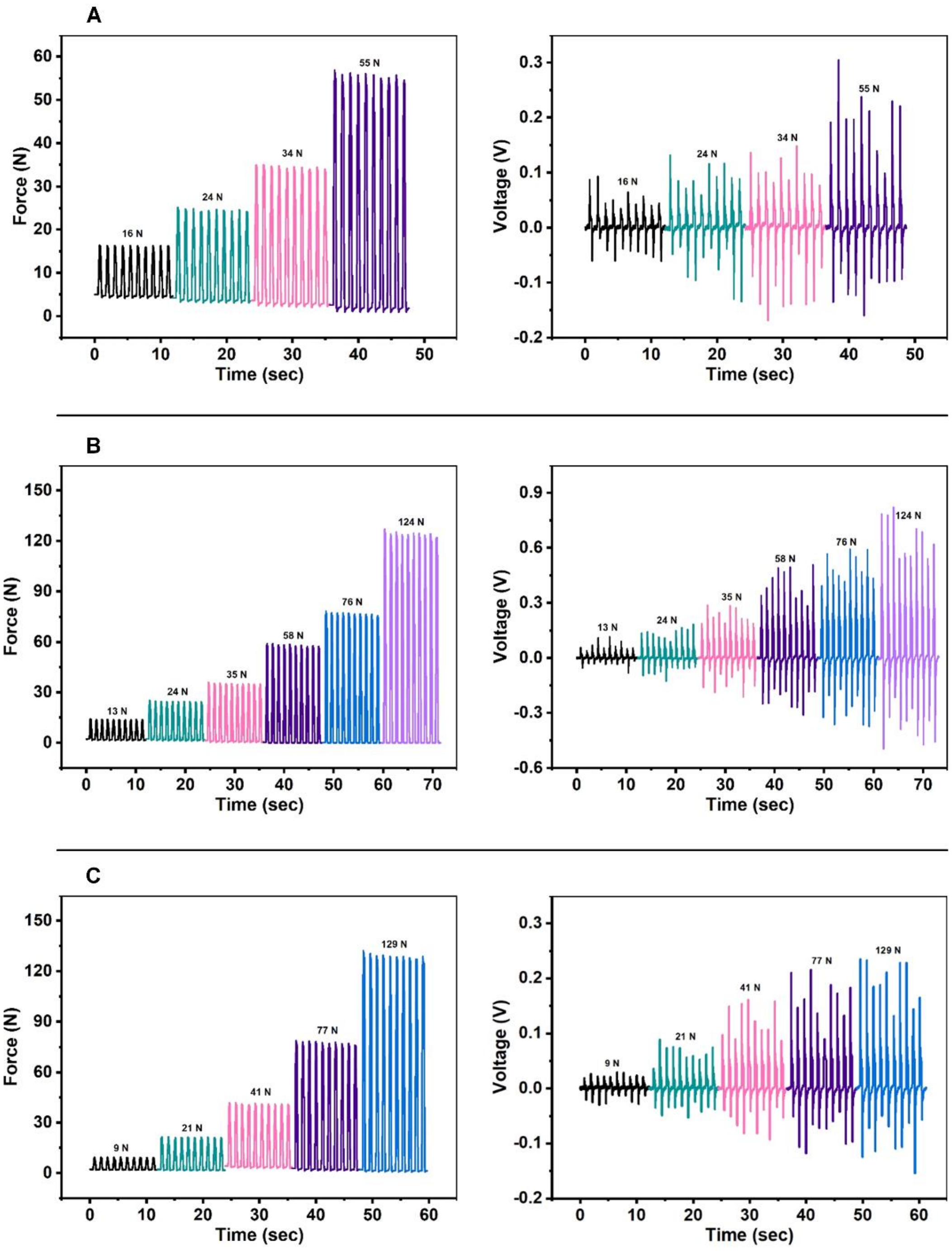

Figure S8. Piezoelectric open-circuit voltage of the PVA/DL-alanine polycrystals according to a periodic compression, related to Figure 5 and STAR Methods. Piezoelectric open-circuit voltage of the PVA/DL-alanine polycrystal with an r of (A) 1, (B) 2, and (C) 3. The force applied to the polycrystals is presented together. All polycrystals were combined with tribo-prevention layer proposed [S1].

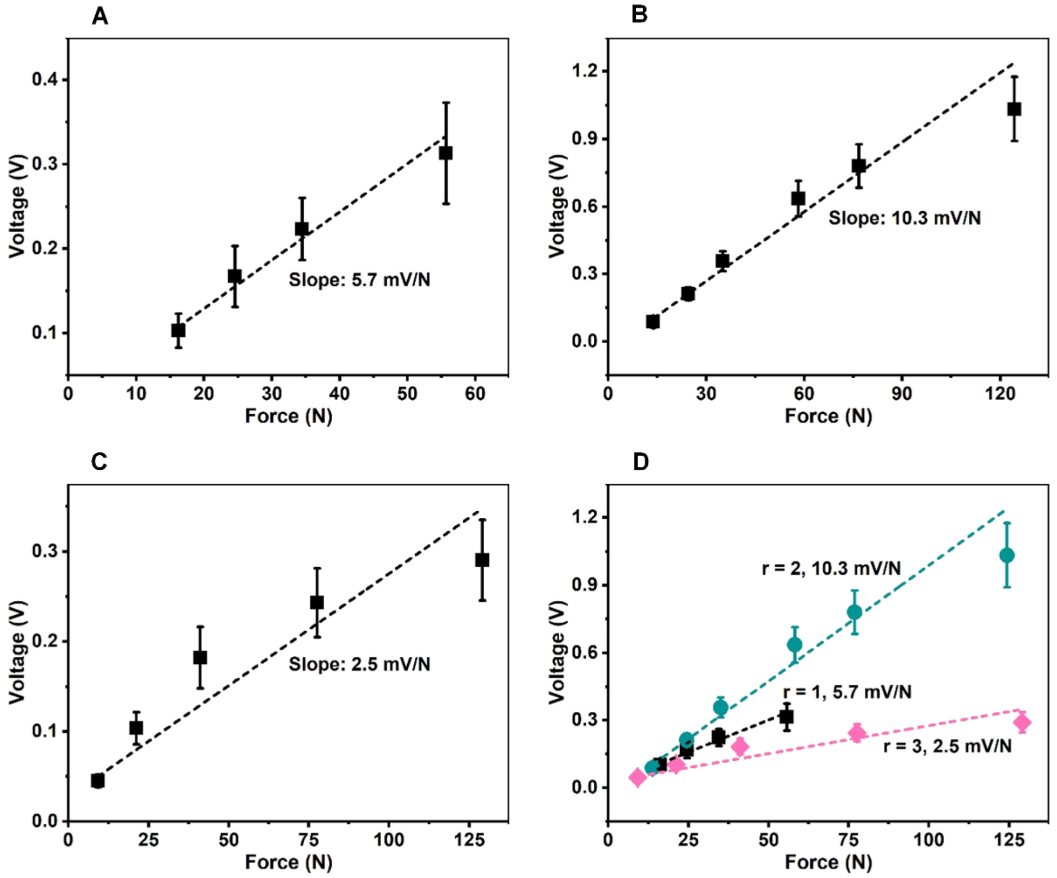

Figure S9. Correlation between the piezoelectric open-circuit voltage and the force of the free-standing PVA/DL-alanine polycrystals, related to Figure 5 and STAR Methods. Correlation between the piezoelectric open-circuit voltage and the force of the free-standing PVA/DL-alanine polycrystal with an r of (A) 1, (B) 2, and (C) 3. (D) Comparison of the correlation between the piezoelectric open-circuit voltage and the force of the free-standing PVA/DL-alanine polycrystals.

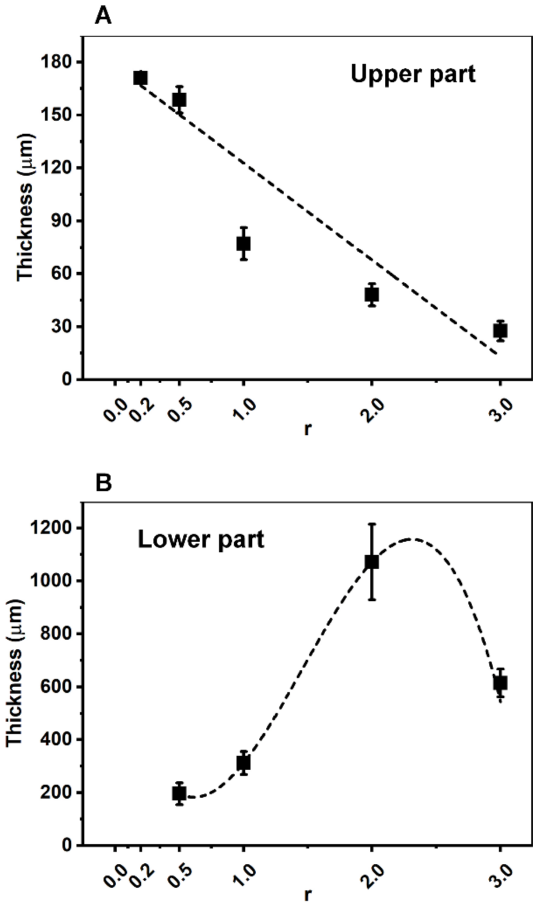

Figure S10. Change of the thickness of the upper and the lower parts of the bilayer structure of the PVA/DL-alanine polycrystals, related to STAR Methods. (A) Thickness change of the upper part of the PVA/DL-alanine polycrystals with different r. (B) Thickness change of the lower part of the PVA/DL-alanine polycrystals with different r.

Table S1. Peak deconvolution of the top surface XRD patterns of the PVA/DL-alanine polycrystals, related to Figure 3. Intensity, crystallinity and FWHM (full width at half maximum) of the diffraction peaks of the XRD patterns are summarized. PVA and DL-ala. indicate that the corresponding peaks come from the polyvinyl alcohol and DL-alanine, respectively. [a,b]Peak splitting of the diffraction peak representing the (400) face of DL-alanine.

Peak position	PVA/DL-alanine polycrystal, r of 0.2			
	Crystal face	Intensity (a.u.)	Crystallinity	FWHM
19.5 °	Amorphous	491.6	46.2 %	6.97 °
19.7 °	(10-1) / PVA	1085.9	24.5 %	1.67 °
20.9 °	(101) / PVA	1541.0	14.9 %	0.72 °
22.7 °	(200) / PVA	190.6	5.9 %	2.31 °
29.7 °	(400) / DL-ala.	304.4	3.5 %	0.85 °
31.0 °	(311) / DL-ala.	202.3	1.3 %	0.49 °
33.3 °	(410) / DL-ala.	334.1	3.7 %	0.82 °
Peak position	PVA/DL-alanine polycrystal, r of 0.5			
	Crystal face	Intensity (a.u.)	Crystallinity	FWHM
14.3 °	(200) / DL-ala.	204.9	18.2 %	0.20 °
16.0 °	(001) / PVA or (110) / DL-ala.	93.3	9.0 %	0.22 °
19.5 °	Amorphous	23.8	25.6 %	2.45 °
20.3 °	(210) / DL-ala.	264.7	28.0 %	0.24 °
29.2 °	(400) / DL-ala.	32.3	2.3 %	0.16 °
30.3 °	(002) / DL-ala.	30.7	3.7 %	0.28 °
32.9 °	(410) / DL-ala.	67.3	13.2 %	0.44 °
Peak position	PVA/DL-alanine polycrystal, r of 1			
	Crystal face	Intensity (a.u.)	Crystallinity	FWHM
14.5 °	(200) / DL-ala.	180.4	0.8 %	0.20 °
15.2 °	(001) / PVA	298.4	2.5 %	0.38 °
16.2 °	(110) / DL-ala.	381.9	2.5 %	0.29 °
20.1 °	Amorphous	234.9	9.4 %	1.76 °
20.6 °	(210) / DL-ala.	8097.0	50.6 %	0.28 °
26.4 °	(310) / DL-ala.	142.1	1.0 %	0.32 °
29.4 °	(400) / DL-ala.	1931.9	14.5 %	0.33 °
30.5 °	(002) / DL-ala.	182.7	1.6 %	0.38 °
32.9 °	(410) / DL-ala.	1668.1	14.1 %	0.37 °
36.9 °	(221) / DL-ala.	172.5	2.9 %	0.75 °
Peak position	PVA/DL-alanine polycrystal, r of 2			
	Crystal face	Intensity (a.u.)	Crystallinity	FWHM
14.6 °	(200) / DL-ala.	221.8	0.7 %	0.33 °

16.3 °	(110) / DL-ala.	2258.9	5.4 %	0.24 °
19.9 °	Amorphous	292.5	5.1 %	1.76 °
20.7 °	(210) / DL-ala.	19017.7	60.3 %	0.32 °
25.7 °	(211) / DL-ala.	172.5	0.6 %	0.34 °
26.6 °	(310) / DL-ala.	410.3	1.3 %	0.33 °
29.6 °	(400) / DL-ala.	964.2	2.9 %	0.31 °
30.6 °	(002) / DL-ala.	533.3	2.2 %	0.4 °
33.1 °	(410) / DL-ala.	4116.8	14.8 %	0.36 °
34.7 °	(112) / DL-ala.	829.0	2.3 %	0.28 °
36.5 °	(221) / DL-ala.	231.1	1.0 %	0.43 °
37.2 °	(320) / DL-ala.	816.8	3.3 %	0.41 °

Peak position	PVA/DL-alanine polycrystal, r of 3			
	Crystal face	Intensity (a.u.)	Crystallinity	FWHM
14.6 °	(200) / DL-ala.	1072.5	3.2 %	1.50 °
16.3 °	(110) / DL-ala.	835.2	3.4 %	0.22 °
19.5 °	Amorphous	235.0	4.7 %	0.31 °
20.7 °	(210) / DL-ala.	6407.0	44.7 %	0.52 °
25.7 °	(211) / DL-ala.	444.1	1.4 %	0.24 °
26.5 °	(310) / DL-ala.	122.7	0.5 %	0.34 °
29.3 ° [a]	(400) / DL-ala.	4239.2	9.0 %	0.16 °
29.6 ° [b]		2204.4	7.6 %	0.36 °
30.5 °	(002) / DL-ala.	1006.5	4.8 %	0.44 °
33.0 °	(410) / DL-ala.	2299.3	13.4 %	0.22 °
34.8 °	(112) / DL-ala.	1802.1	5.2 %	0.38 °
36.5 °	(221) / DL-ala.	163.9	0.8 %	0.39 °
37.1 °	(320) / DL-ala.	241.1	1.2 %	0.26 °

Table S2. Peak deconvolution of the XRD patterns measured at the bottom surface of the PVA/DL-alanine polycrystals, related to Figure 3. Intensity, crystallinity and FWHM (full width at half maximum) of the diffraction peaks of the XRD patterns are summarized. PVA and DL-ala. indicate that the corresponding peaks come from the polyvinyl alcohol and DL-alanine, respectively.

Peak position	PVA/DL-alanine polycrystal, r of 0.2			
	Crystal face	Intensity (a.u.)	Crystallinity	FWHM
14.7 °	(200) / DL-ala.	625.5	3.5 %	0.29 °
16.3 °	(110) / DL-ala.	523.66	3.8 %	0.38 °
19.7 °	Amorphous or (10-1) / PVA	1208.6	46.9 %	2.06 °
20.8 °	(210) / DL-ala.	5679.8	33.0 %	0.31 °
29.6 °	(400) / DL-ala.	1196.6	7.0 %	0.31 °
33.2 °	(410) / DL-ala.	771.6	5.9 %	0.40 °
Peak position	PVA/DL-alanine polycrystal, r of 0.5			
	Crystal face	Intensity (a.u.)	Crystallinity	FWHM
14.5 °	(200) / DL-ala.	278.3	3.0 %	0.39 °
16.3 °	(110) / DL-ala.	758.1	6.3 %	0.30 °
19.8 °	Amorphous or (10-1) / PVA	286.0	13.0 %	1.63 °
20.7 °	(210) / DL-ala.	5147.9	40.7 %	0.28 °
21.1 °	(201), (011) / DL-ala.	1159.6	5.4 %	0.17 °
25.8 °	(211) / DL-ala.	411.7	2.5 %	0.22 °
29.6 °	(400) / DL-ala.	385.9	4.0 %	0.37 °
30.7 °	(002) / DL-ala.	1472.4	11.1 %	0.27 °
33.1 °	(410) / DL-ala.	906.5	8.2 %	0.32 °
36.6 °	(221) / DL-ala.	494.0	4.2 %	0.30 °
37.2 °	(320)/ DL-ala.	159.8	1.7 %	0.37 °
Peak position	PVA/DL-alanine polycrystal, r of 1			
	Crystal face	Intensity (a.u.)	Crystallinity	FWHM
14.5 °	(200) / DL-ala.	525.1	2.2 %	0.26 °
16.2 °	(110) / DL-ala.	1097.2	4.7 %	0.27 °
19.7 °	Amorphous or (10-1) / PVA	492.3	15.6 %	2.02 °
20.7 °	(210) / DL-ala.	8170.8	38.6 %	0.30 °
21.1 °	(201), (011) / DL-ala.	2403.6	8.2 %	0.22 °
25.8 °	(211) / DL-ala.	242.2	0.96 %	0.25 °
26.5 °	(310) / DL-ala.	182.1	0.8 %	0.29 °
29.5 °	(400) / DL-ala.	1232.1	5.4 %	0.28 °
30.7 °	(002) / DL-ala.	1044.4	4.9 %	0.30 °

33.0 °	(410) / DL-ala.	2104.7	12.5 %	0.38 °
34.9 °	(112) / DL-ala.	672.7	2.9 %	0.28 °
36.6 °	(221) / DL-ala.	210.6	1.2 %	0.36 °
37.2 °	(320) / DL-ala.	365.2	2.2 %	0.39 °

Peak position	PVA/DL-alanine polycrystal, r of 2			
	Crystal face	Intensity (a.u.)	Crystallinity	FWHM
14.5 °	(200) / DL-ala.	294.4	1.8 %	0.36 °
16.3 °	(110) / DL-ala.	322.9	2.7 %	0.48 °
19.8 °	Amorphous or (10-1) / PVA	481.4	26.8 %	3.25 °
20.7 °	(210) / DL-ala.	3004.9	18.8 %	0.37 °
21.1 °	(201), (011) / DL-ala.	4787.2	17.7 %	0.22 °
25.8 °	(211) / DL-ala.	320.6	1.0 %	0.19 °
29.5 °	(400) / DL-ala.	727.4	4.5 %	0.36 °
30.7 °	(002) / DL-ala.	2071.8	10.5 %	0.30 °
33.1 °	(410) / DL-ala.	541.0	4.0 %	0.43 °
34.9 °	(112) / DL-ala.	1947.5	8.7 %	0.26 °
37.2 °	(320) / DL-ala.	776.5	3.5 %	0.26 °

Peak position	PVA/DL-alanine polycrystal, r of 3			
	Crystal face	Intensity (a.u.)	Crystallinity	FWHM
14.5 °	(200) / DL-ala.	735.0	3.3 %	0.32 °
16.3 °	(110) / DL-ala.	1684.0	7.6 %	0.33 °
20.3 °	Amorphous or (10-1) / PVA	796.3	15.2 %	1.38 °
20.7 °	(210) / DL-ala.	7582.5	43.0 %	0.41 °
21.1 °	(201), (011) / DL-ala.	774.3	1.9 %	0.18 °
25.7 °	(211) / DL-ala.	159.4	0.6 %	0.28 °
26.5 °	(310) / DL-ala.	162.9	0.6 %	0.28 °
29.5 °	(400) / DL-ala.	1858.5	8.3 %	0.33 °
30.5 °	(002) / DL-ala.	1197.1	5.4 %	0.33 °
33.1 °	(410) / DL-ala.	1092.1	8.6 %	0.57 °
34.8 °	(112) / DL-ala.	640.9	2.5 %	0.28 °
36.5 °	(221) / DL-ala.	344.0	1.6 %	0.34 °
37.0 °	(320) / DL-ala.	199.3	1.4 %	0.52 °

REFERENCE

1. Chen, C., Zhao, S., Pan, C., Zi, Y., Wang, F., Yang, C., & Wang, Z. L. (2022). A method for quantitatively separating the piezoelectric component from the as-received "Piezoelectric" signal. Nature communications, 13(1), 1-9. DOI: https://doi.org/10.1038/s41467-022-29087-w.
